# Single-shot super-resolved fringe projection profilometry (SSSR-FPP): 100,000 frames-per-second 3D imaging with deep learning

**DOI:** 10.1038/s41377-024-01721-w

**Published:** 2025-02-07

**Authors:** Bowen Wang, Wenwu Chen, Jiaming Qian, Shijie Feng, Qian Chen, Chao Zuo

**Affiliations:** 1 Smart Computational Imaging Laboratory (SCILab), Nanjing University of Science and Technology, Nanjing, Jiangsu Province China; 2 Jiangsu Key Laboratory of Spectral Imaging & Intelligent Sense, Nanjing University of Science and Technology, Nanjing, Jiangsu Province China

**Keywords:** Optical metrology, Imaging and sensing

## Abstract

To reveal the fundamental aspects hidden behind a variety of transient events in mechanics, physics, and biology, the highly desired ability to acquire three-dimensional (3D) images with ultrafast temporal resolution has been long sought. As one of the most commonly employed 3D sensing techniques, fringe projection profilometry (FPP) reconstructs the depth of a scene from stereo images taken with sequentially structured illuminations. However, the imaging speed of current FPP methods is generally capped at several kHz, which is limited by the projector-camera hardware and the number of fringe patterns required for phase retrieval and unwrapping. Here we report a novel learning-based ultrafast 3D imaging technique, termed single-shot super-resolved FPP (SSSR-FPP), which enables ultrafast 3D imaging at 100,000 Hz. SSSR-FPP uses only one pair of low signal-to-noise ratio (SNR), low-resolution, and pixelated fringe patterns as input, while the high-resolution unwrapped phase and fringe orders can be deciphered with a specific trained deep neural network. Our approach exploits the significant speed gain achieved by reducing the imaging window of conventional high-speed cameras, while “regenerating” the lost spatial resolution through deep learning. To demonstrate the high spatio-temporal resolution of SSSR-FPP, we present 3D videography of several transient scenes, including rotating turbofan blades, exploding building blocks, and the reciprocating motion of a steam engine, etc., which were previously challenging or even impossible to capture with conventional methods. Experimental results establish SSSR-FPP as a significant step forward in the field of 3D optical sensing, offering new insights into a broad spectrum of dynamic processes across various scientific disciplines.

## Abstract (web summary)

We present a learning-based ultrafast 3D imaging technique, termed single-shot super-resolved FPP (SSSR-FPP), that is capable of reconstructing 3D images of non-repetitive dynamic events at 100,000 frames per second (fps).

## Graphical abstract

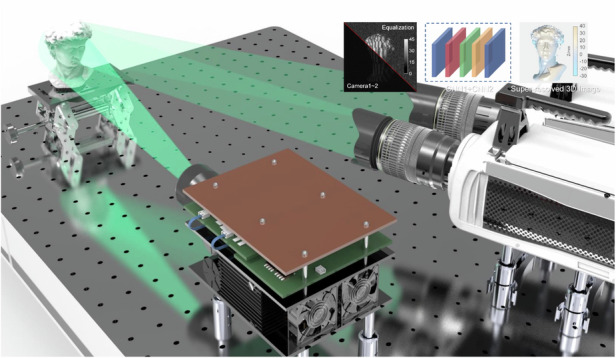

## Introduction

The ability to probe fast-occurring events in three dimensions (3D) with ultrafast temporal resolution has been of vital importance for gaining new insight and understanding fundamental scientific questions in mechanics, physics, and biology^1–3^. Current charge-coupled device (CCD)- and complementary metal-oxide-semiconductor (CMOS)-based image sensors only record two-dimensional (2D) image sequences that lack depth information, and their imaging frame rates only reach a few kHz at a decent resolution^4–6^. In addition, due to the limited speed of data transfer and sensor integration time, increasing the frame acquisition rate often results in a significant reduction in imaging resolution and signal-to-noise ratio (SNR).

With the advances in electronic imaging sensors, we have witnessed the rapid evolution of 3D image acquisition technologies over the past decades^7,8^. As one of the most widely adopted 3D sensing techniques, fringe projection profilometry (FPP) reconstructs the depth information of a scene from stereo images taken with sequential structured illuminations^9–12^. However, for measuring dynamic or even transient events, the imaging speed of FPP is capped by two fundamental factors: (1) hardware: the speed of the projector and camera; and (2) software (algorithm): the number of patterns required per 3D reconstruction. These two factors are complementary to make 3D imaging “faster”: one should employ high-speed hardware while simultaneously reducing the number of fringe patterns needed for the 3D reconstruction^13^.

In terms of “hardware”, binary defocusing techniques have been explored to produce quasi-sinusoidal fringes with 1-bit binary patterns through defocusing of the projector lens^14–16^. These methods take advantage of the binary operation mechanism inherent in digital-light-processing (DLP) technology to break the speed bottleneck of conventional FPP techniques that typically employ 8-bit sinusoidal fringe patterns, permitting tens of kHz fringe projection by utilizing a digital micromirror device (DMD)^13,17,18^. On the other hand, various pattern schemes and decoding algorithms for absolute phase retrieval have been developed, e.g., dual-frequency phase shifting^19^, bi-frequency phase shifting^20^, 2+2 phase shifting^21^, geometric constraints-based composite phase-shifting method^22^, speckle-embedded Fourier transform algorithm^23^, and micro Fourier transform profilometry^13^. These methods remove the encoding redundancy in the traditional Gray code or multi-frequency phase-shifting methods, effectively reducing the number of patterns required for unambiguous 3D reconstruction. Despite these significant advancements, extracting high-precision absolute phase information from one single fringe pattern remains a challenge.

With the advancements in artificial intelligence, particularly deep learning (DL)^24–26^, optical metrology — a field dedicated to the precise measurement and characterization based on optical signals, has experienced a paradigm shift. The advantage of DL lies in its ability to automatically extract features^27–30^ and patterns from a large amount of data, thereby offering novel data-driven solutions to various complex problems in optical metrology. Nowadays, deep learning has gradually “permeated” into almost all facets of optical metrology^31^, e.g., fringe analysis^32–34^, fringe denoising^35–37^, and phase unwrapping^38–41^. In particular, it has been demonstrated that properly trained deep neural networks (DNNs) can retrieve phase and unambiguous 3D coordinates of complex objects from only a single fringe pattern, effectively pushing the 3D imaging speed to align with the camera’s native frame rate for 2D image acquisition. Nevertheless, as mentioned earlier, the high-speed cameras currently available can only achieve an imaging frame rate of a few kHz with a decent resolution. Consequently, the highest 3D imaging frame rate of reported FPP techniques only reaches ~ 20 kHz^42^, which still falls short of the requirements for capturing ultra-fast phenomena.

**Fig. 1:**
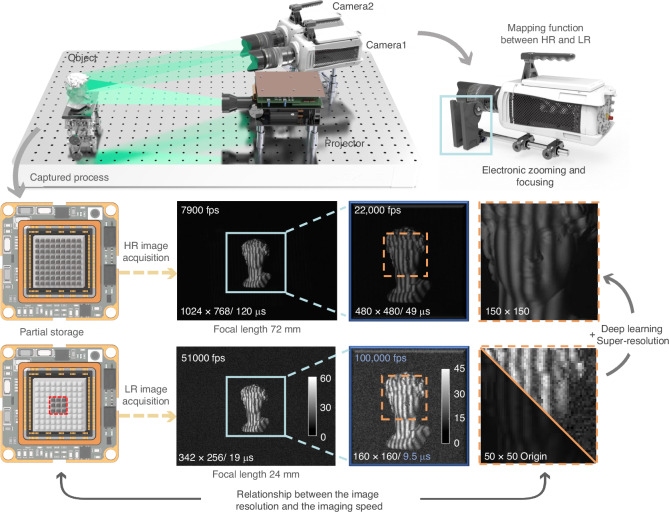
Schematic diagram of the SSSR-FPP system. The SSSR-FPP system includes two high-speed CMOS scientific cameras (Vision Research Phantom V611) and a customized DLP projection system. With the increase in a camera’s maximum frame rate, the exposure time is too limited to capture a sufficiently bright image, resulting in images with poor SNR and evident read noise superimposed

In this work, we report a novel learning-based ultrafast 3D imaging method, termed single-shot super-resolved FPP (SSSR-FPP), as shown in Fig. 1, that is capable of reconstructing 3D images of non-repetitive dynamic events at 100,000 frames per second (fps). It uses only one pair of low-SNR, low-resolution (LR), pixelated fringe patterns as input, while the high-resolution (HR) unwrapped phase and fringe orders can be deciphered with a specifically trained network. The novelty of the SSSR-FPP concept lies in exploiting the significant speed gain achieved by reducing the imaging window of conventional high-speed cameras, while the lost spatial resolution is then regenerated with deep learning. As a result, we reveal the potential of combining deep learning with FPP for ultrafast, super-resolved, ambiguity-free 3D imaging, pushing the 3D imaging frame rate into the 100 kHz regime. In the following sections, we will outline the principle of SSSR-FPP and its experimental setup, quantify its super-resolution performance and 3D measurement accuracy, and demonstrate its applications in various transient scene categories.

## Results

By leveraging the substantial speed gained from reducing the imaging window of conventional high-speed cameras, SSSR-FPP attempts to retrieve a high-SNR and high-quality 3D image from a pair of single low-SNR, low-resolution fringe patterns. As depicted in Fig. 1, the SSSR-FPP system comprises two high-speed scientific cameras (Vision Research Phantom V611) and a customized DLP projection system equipped with an XGA resolution (1024 × 768) DMD chip. The cameras operate within a localized readout window of 160 × 160 pixels, enabling them to capture consecutive images at a frame rate of 100,000 fps with a maximum exposure time of 9.5 *μ*s (see Supplementary Information, Section B for more details about the hardware system).

**Fig. 2:**
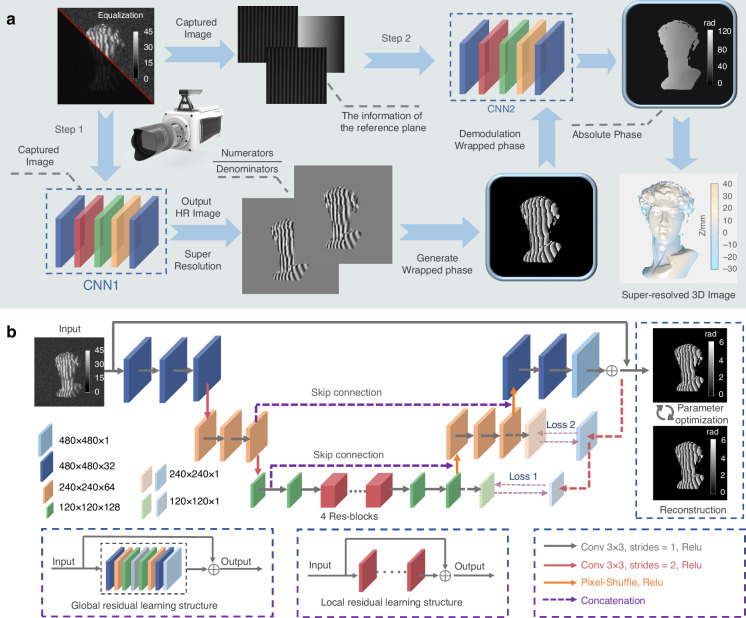
The workflow of the proposed SSSR-FPP method. **a** Step 1: input the LR gray fringe patterns, and output the numerator and denominator terms. Step 2: generate the predicted high-accuracy absolute phase and then recover the 3D shape by the pre-calibration parameters. **b** The network structure of our method, which employs an encoder-decoder structure. Skip connection enhances the transmission of image feature information while alleviating the problem of gradient disappearance. Considering the asymmetric mapping of learning, an additional constraint is deployed, which can be regarded to mutually retain the dual-regression mapping between HR and LR images to enforce the network robustness and the generalization capability under real-world settings

The primary challenge of SSSR-FPP lies in deciphering absolute phase distribution with decent resolution and SNR from only a single pair of low-quality, pixelated fringe images. Inspired by the recent success of DNN applied in image super-resolution^43^, fringe analysis^32,44,45^, and geometric phase unwrapping^31,40^, we propose to leverage deep learning to address this challenge. As schematically described in Fig. 2, SSSR-FPP employs two structurally similar but functionally different convolutional neural networks (CNNs), CNN1 and CNN2, which work in concert to achieve both super-resolved phase retrieval and phase unwrapping (detailed in Supplementary Information, Section A). A low-resolution (160 × 160) fringe image is the input of CNN1, which is integrated with the traditional phase-shifting physical model to generate the 3 × super-resolved (480 × 480) numerator (sine) and denominator (cosine) components of the wrapped phase function. These two components are then jointly processed through the arctangent function to predict a high-resolution wrapped phase map. The proposed approach circumvents the challenges associated with accurately following the 2*π* phase jumps by directly employing an end-to-end network structure, thereby improving the accuracy of the reconstructed phase effectively. CNN2 is designed to predict a low-precision absolute phase from the input fringe image. Though the absolute phase output by CNN2 is relatively “coarse”, it is adequate to resolve the fringe order of the high-precision, high-resolution wrapped phase predicted by CNN1 so that the high-precision absolute phase can be obtained. Based on the pre-calibrated geometric parameters of the experimental setup, a high-precision 3D point cloud can be reconstructed, and thus, single-shot super-resolved structured light 3D imaging at a frame rate of 100,000 fps can be realized.

### Measurement results of a static plaster model

**Fig. 3:**
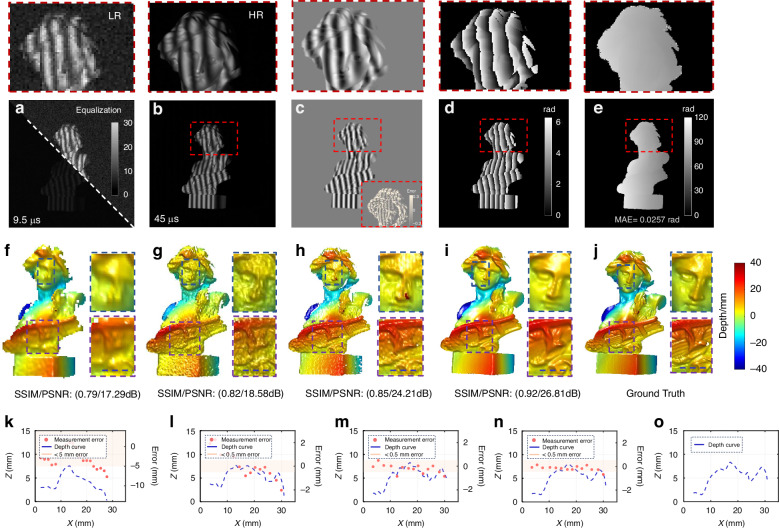
Static plaster model reconstruction results. **a** Low-resolution fringe pattern (The raw image that is fed into the network). **b** High-resolution fringe pattern (Ground truth image of the network). **c** The predicted numerator. **d** The predicted wrapped phase. **e** The predicted absolute phase. **f** Reconstruction results were produced using a 3-frequency and 3-step phase-shifting method with the raw low-resolution image. **g** Reconstruction results acquired with a 3-frequency and 3-step phase-shifting approach using the bicubic interpolation image. **h** Reconstruction results generated by a 3-frequency and 12-step phase-shifting method using the bicubic interpolation image. **i** 3D reconstruction obtained by our method. **j** Ground truth (High-resolution 3-frequency and 12-step phase-shifting unwrapping method result, denoted as the ground truth). **k**–**o** Horizontal profiles (x-z) of 3D reconstruction results (The blue dot line shown in the magnified zoom region of figure **f**–**j**) under different methods with the disparity to the truth values

To evaluate the super-resolution capability of the proposed SSSR-FPP, we measured a static plaster model, which was not included in the dataset used for training our neural networks. Due to the short exposure time (9.5 *μ*s) and the low pixel resolution (160 × 160 pixels), background noise and mosaic effect are evident in the raw images captured at 100,000 fps (refer to Fig. 3a and the corresponding zoomed region). The corresponding ideal high-resolution fringe image of 480 × 480 pixels, captured at a focal length of 72 mm, is shown in Fig. 3b, where noise and pixelation are mitigated at the expense of a much lower frame rate (22,000 fps) and longer exposure time (45 *μ*s). Without altering the hardware setup, the trained CNN1 takes the low-resolution fringe pattern as input and predicts the corresponding super-resolved background-free fringe amplitude image. Image resolution and fringe quality are improved significantly, as verified by the output numerator term (sine component of the ideal fringe image) shown in Fig. 3c, with the error from the ground truth displayed in the lower right corner. Following the output from CNN1, the high-resolution wrapped phase is calculated using the arctangent function, as shown in Fig. 3d. The wrapped phase, along with the reference images, is then fed into CNN2 to produce the high-resolution phase distribution, as depicted in Fig. 3e. The mean absolute phase error (MAE) of the proposed method is only 0.0257 rad for the measured scenario involving complex surfaces.

To further verify the super-resolution 3D imaging capability, we compared the 3D reconstruction results obtained by our method with those generated from raw low-resolution fringe patterns using various up-sampling strategies. It should be noted that these conventional methods still require 3-frequency and 3-step phase-shifting algorithm to determine the absolute phase, entailing a total of nine fringe patterns. The 3D-rendered geometries, converted from the unwrapped phase maps through stereo triangulation, offer a more intuitive comparison, as shown in Fig. 3f–j. Two areas (boxed regions) containing fine structures are enlarged and shown on the right, with the bottom row showing the line profile along the blue dotted line. As shown in Fig. 3f, the reconstruction from raw low-resolution data is too coarse to discern the model’s facial features and clothing details. While up-sampling of the raw fringe images yielded a higher quality 3D reconstruction that preserves surface details more effectively, they suffered from periodic phase errors stemming from non-sinusoidal waveforms (a consequence of inaccurate interpolation), as can be seen in Fig. 3h. In contrast, the proposed SSSR-FPP method produced the most accurate 3D reconstruction, with fine surface details well-captured, closely mirroring the ground truth data obtained from high-resolution (480 × 480 pixels) 3-frequency and 12-step phase-shifted fringe images. A comparative analysis of the depth profiles for a selected area is further presented in Fig. 3k–o. Our method faithfully reproduced the shape and features present in the ground truth, exhibiting a high degree of structural similarity in both depth values and the precise identification of inflection points. The depth measurement error curve indicates that the error between the network output and the true value is less than 0.5 mm. Objective quantitative assessment based on the Structural Similarity Index (SSIM) and Peak Signal-to-Noise Ratio (PSNR) metrics further validated the fidelity and high SNR of our reconstruction, with values of 0.92 and 26.81 dB, respectively. In contrast, due to pixel aliasing and noise interference, the error distributions of other methods significantly exceed 0.5 mm. Specifically, when compared with the non-interpolated 3-frequency and 3-step phase-shifting methods, our approach demonstrated substantial improvement, with an increase of 0.13 in SSIM and 9.52 dB in PSNR.

To demonstrate the rationality and effectiveness of the constructed deep learning network, we employed the same training datasets and compared the performance of different network structures (MVSNet^46^, DCNN^47^, Multi-path CNN^32^, etc.) with our method in a comparative experiment in Fig. S9. Experimental results show that the constructed network can preserve the smallest MAE (0.0871 rad) in the predicted absolute phase, especially in the region of sharp edges or significant variations in reflectivity, and thus guarantee the highest precision of ultra-high-speed 3D imaging and measurement. More details can be found in Section D of the Supplementary Information. In addition, to further demonstrate the generalization capability of the SSSR-FPP method, in Section F of the Supplementary Information, we present additional comparative experimental results of four different plaster models. These results confirm that SSSR-FPP is robust against challenges such as low image SNR and can be reliably applied to a variety of samples with complex shapes. It is important to note that the SSSR-FPP reconstruction is fully automated and does not require manual adjustment of parameters, thereby enhancing its algorithmic efficiency. In consideration of the aforementioned discussions, the SSSR-FPP method offers a promising solution for single-shot 3D super-resolved reconstruction, minimizing the impact of phase measurement errors at low SNR to the greatest extent possible with current technology.

### Quantitative analysis of 3D reconstruction accuracy

**Fig. 4:**
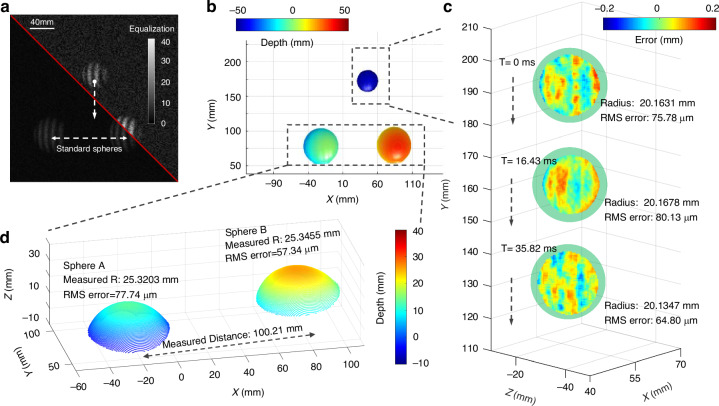
Accuracy analysis of 3D reconstruction using the SSSR-FPP Method. **a** The test scenario consists of a standard ceramic ball and a free-falling table tennis ball, both the raw image and the equalized image are shown. **b** The color-coded rendering of the 3D reconstruction result. **c** Error distribution of free-falling table tennis in three discrete moments (T = 0 ms, 16.43 ms, 35.82 ms). **d** The error distributions of enlarged areas corresponding to the ceramic balls

To quantitatively evaluate the accuracy of SSSR-FPP measurement results, we further conducted a dynamic measurement scenario involving a pair of standard ceramic spheres and a table tennis ball in free fall. The standard spheres have certified radii of 25.3989 mm and 25.4038 mm, respectively, with a center-to-center distance of 100.0532 mm. This allows us to gauge the measurement precision and repeatability of the SSSR-FPP system using spheres with precisely calibrated dimensions. The table tennis ball, with a radius of approximately 19.8 ± 0.1 mm, was used to test the system’s accuracy to measure moving objects. Figure 4b presents the color-coded 3D shapes of the two standard spheres and the falling table tennis ball measured by the proposed SSSR-FPP method at T = 0 ms. As shown in Fig. 4d, the measurement errors (root mean square) for the standard spheres are 77.74 *μ*m and 57.34 *μ*m, respectively. The measured center-to-center distance between the two spheres is 100.21 mm. In Fig. 4c, we provide the measurement errors for a free-falling ball at three distinct instants (T = 0 ms, 16.43 ms, 35.82 ms). Since the dimension of the table tennis ball was uncalibrated, the accuracy of the measurements was determined by fitting the point cloud and calculating the discrepancy between the measured data and the fitted sphere, and the spherical radius was determined by fitting an optimal sphere to the 3D point cloud. These results demonstrate that SSSR-FPP is capable of performing quantitative absolute 3D shape measurement with an accuracy better than 80 *μ*m within a volume of 260 mm × 260 mm × 50 mm.

### Experimental results of dynamic scenes

**Fig. 5:**
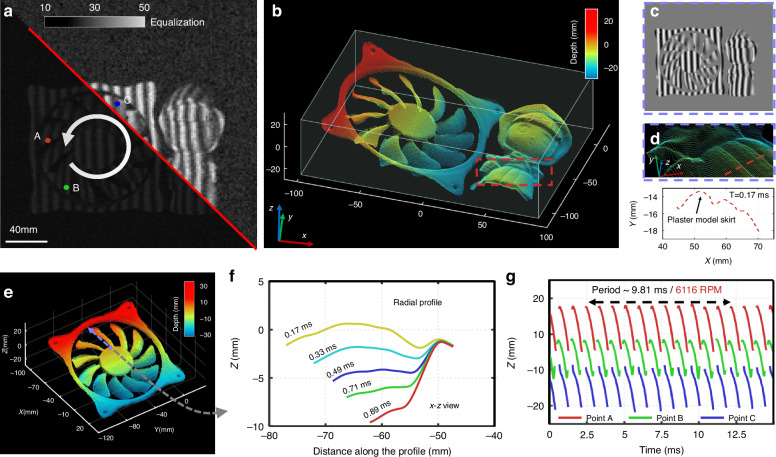
Measurement of rotating blades and a static plaster model. **a** The raw image obtained by the camera. **b** At the onset of the observation period (T = 0 ms), the entire 3D shape of the fan was remodeled, with the inset showing a local zoom of the 3D reconstruction. **c** The numerator predicted by the network. **d** Detailed enlargement and height profile at the red line position in (**b**). **e** The fan’s surface is represented in a color-coded 3D rendering, frozen in time at T = 0.17 ms. **f** Height curves plotted along the radial direction (corresponding to the dashed line in e), each with a corresponding time interval of 0.17 ms. **g** Displacement variation in the z-direction at three picked point locations (A, B, and C, as presented in **a**) over a time function of 15 ms

We demonstrate the high temporal resolution of SSSR-FPP by performing 3D videography of a fast-changing scenario containing two isolated samples: a fast-spinning computer fan and a static plaster model located on the right side. Despite the background noise and pixelation in the raw fringe images, the 3D geometry of the entire fan and the surface details of the plaster model, including the curly hair and the ripples on the skirt, were well-resolved by SSSR-FPP, as demonstrated in Fig. 5b. An enlarged view of the corresponding areas is presented in Fig. 5d. The corresponding depth curves clearly show that the skirt of the plaster model was accurately reconstructed at t = 0.17 ms.

To test the repeatability of the 3D measurement data, we randomly selected three points on the fan blade to illustrate the periodic motion (A, B, and C, marked in Fig. 5a). As shown in Fig. 5g, the depth variations in the z-direction at these selected positions are plotted for a period of 15 ms as a function of time. The graph indicates that the rotation period of the fan is about 9.81 ms, equivalent to a speed of 6,116 revolutions per minute (RPM), demonstrating the stable repeatability of the SSSR-FPP measurement. The color-coded 3D rendering of the fan surface at ~ 0.17 ms is presented in Fig. 5e. Figure 5f demonstrates five line profiles drawn outwards from the central hub along the radial direction. Within the short period of 0.89 ms, the fan blades rotated rapidly, completing about 1/11 of a turn from the initial position, producing a maximum depth variation of over 8 mm in the z-direction. The length of the fan blade can be further estimated from the 3D reconstruction to be ~ 110 mm, with a radius of ~45 mm. The corresponding 3D-rendered video sequence is available in Supplementary Visualization 2.

**Fig. 6:**
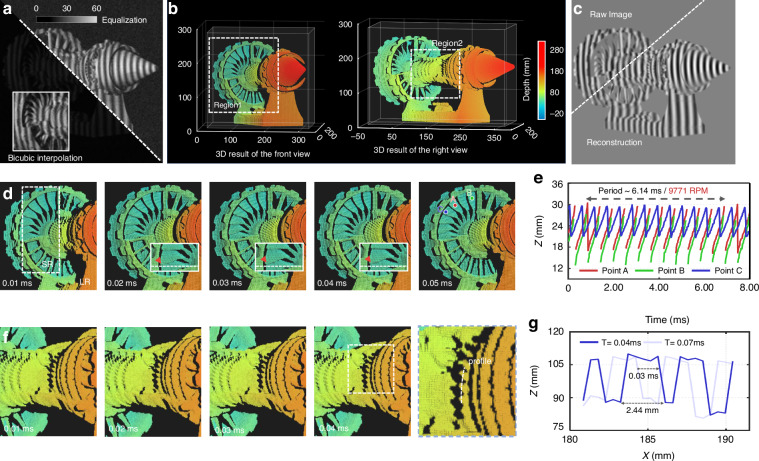
3D measurement of prototype turbofan engine components while operating at the maximum physical rotational speed. **a** The raw image obtained by the camera. **b** 3D rendering of a turbofan engine model from two different viewpoints. **c** The numerator predicted by the network and the corresponding raw image obtained by 3-frequency and 12-step phase-shifting method. **d** 3D rendering of color-coded frozen at different moments for selected region 1. The inset displays the 3D measurement variation with a time interval of 0.01 ms. **e** z-direction displacement fluctuations at the selected points A, B, and C, as illustrated in figure d, are charted over an 8-millisecond timespan. **f** 3D rendering of color-coded frozen at different moments for selected region 2. **g** The cross-sectional profiles along the x-z plane of the gear’s marking curve at two different points, as depicted in figure (**f**)

Finally, we performed 3D reconstruction of a turbofan engine model at its maximum physical rotational speed. The original image captured is presented in Fig. 6a. Despite the high-frequency details in the raw fringe image being distorted by the noise superimposed, SSSR-FPP successfully resolved the textural characteristics, as shown in Fig. 6b. As shown in Fig. 6b, the 3D rendering of the turbofan engine model is displayed from two different perspectives. The numerator (sine) term of the network output is shown in Fig. 6c. To validate the reliability of the SSSR-FPP reconstruction, we selected three arbitrary points on the flywheel, labeled as A, B, and C in Fig. 6d, to showcase the periodic rotation. Figure 6d shows the successive 3D measurement results at a time interval of 0.01 ms. The inset provides a magnified view of the blade region, allowing for an intuitive observation of the surface shape variation (the blade rotated approximately 5 pixel points in the image with an interval of 0.02 ms). In Fig. 6e, we graphed the vertical displacement along the z-axis at the designated points, which were measured over an 8 ms period. It can be observed that the fan’s rotational period is approximately 6.14 ms, corresponding to a speed of 9771 RPM. Figure 6f presents an enlarged view of the selected region 2, providing a closeup perspective that captured the texture details of the gears at various moments. We conducted a cross-sectional analysis in the x-z plane on a specific area of the turbine’s turntable. As shown in Fig. 6g, the measured gear width is approximately 2.44 mm. After 0.03 ms, the forward movement increment of the gear is about half a gear. The analysis confirms that the SSSR-FPP technique is capable of delivering high-resolution ultrafast 3D shape measurements across a measurement volume of 400 mm × 180 mm × 210 mm. The reconstructed 3D-rendered video sequence over an observation period of 6.14 ms is further provided in Supplementary Visualization 3.

In addition to the three dynamic experiments demonstrated above, we have also presented additional high-speed 3D imaging results across diverse scenarios in Supplementary Information, Section G. These additional experiments include capturing non-repetitive transient events such as a flying bullet, analyzing the physical transformation process of a steam engine, recording complex object deformations, and measuring objects with diverse materials and colors. The corresponding 3D video sequences can be found in Supplementary Visualization 4–8. These supplementary experimental results further consolidate the robustness and universality of the high-speed dynamic imaging capability of SSSR-FPP, particularly for measuring fast-moving objects with complex geometries and surface reflectivities, at an unprecedented speed of 100 kHz.

## Discussion

### Analysis of spatial resolution and SNR of the SSSR-FPP system

The intrinsic restriction on the maximum frame rate of a high-speed camera is mainly determined by the time required to read out the captured images from the detector, which is determined by the readout speed, pixel clock rate, and the dimension of the image to be read out (i.e., the pixel resolution). As a result, most high-speed cameras can achieve significantly higher frame rates by substantially reducing the frame size, typically achieved by averaging or windowing the neighboring pixels and performing image recording and readout for a subset of the sensor’s full area.

Taking the commercially available high-speed camera used in our SSSR-FPP system, Phantom V611, as an example, its maximum read-out speed is ~ 6 Gigapixels per second, corresponding to only 6224 fps at full megapixel resolution (1280 × 800). However, it is capable of acquiring over 20,000 fps with about a quarter of the full size (512 × 512), 100,000 fps with a significantly reduced resolution (160 × 160), and achieving a maximum speed of 1000 kHz with only a few lines of pixels (128 × 8). In addition to the reduction in image resolution, there exists a direct relationship between the camera’s frame rate and its exposure time (integration time), which in turn directly affects the imaging sensitivity and SNR. As an example shown in Fig. 1, the maximum attainable frame rate cannot surpass the physical limit governed by the sensor exposure time. With the increase in camera’s frame rate, the exposure time becomes too short to acquire sufficient photons, resulting in images with a poor SNR and pronounced readout noise.

In the realm of high-speed imaging, it is common practice for high-speed imaging devices to utilize image sensors with large pixels to guarantee adequate detection sensitivity. A larger pixel size offers higher light-gathering capabilities, albeit at the expense of reduced spatial resolution due to lower sampling density. The imaging resolution of most high-speed cameras is primarily constrained by the Nyquist sampling limit determined by pixel size, rather than the diffraction limit imposed by the imaging optics. Therefore, achieving an optimal balance between sensitivity and resolution is essential in designing high-speed imaging systems. This balance allows for capturing high-quality images while maintaining an accurate representation of fine details, even under challenging conditions of high-speed imaging with limited exposure time.

The proposed imaging system utilizes a 20 *μ*m pixel size sensor (Phantom CMOS), coupled with a 24 mm focal length lens, which theoretically enabled the system to achieve a spatial resolution of 0.833 mrad. Through SSSR-FPP reconstruction, the spatial resolution can be enhanced up to 0.891 lp/mm (Group -1, Element 6), corresponding to a 1.58 × improvement in resolution. Leveraging the prior knowledge embedded in the network significantly improves the spatial resolution, rendering the texture details on the plaster models sharper, in conjunction with the improvement of SNR from 28.57 dB to 33.51 dB (further discussions about the spatial resolution and the accuracy of the reconstructed phase distribution can be found in Supplementary Information, Section D). It is worth noting the proposed method offers higher accuracy in both lateral and axial directions than conventional solutions, providing a low-cost yet robust solution for high-speed 3D imaging.

### Advantages of SSSR-FPP for ultrafast 3D imaging

Furthermore, we conducted a comparative experiment to analyze the influence of different fringe pattern schemes on the reconstruction results under ultra-high-speed imaging conditions. We find that the composite fringes-based 3D imaging methods become vulnerable under pixelated and low SNR sampling conditions, due to the fact that the reduced resolution and SNR make the problem of spectral aliasing more severe, which is fatal in techniques like multiplexed FPPs. (Details are in the Supplementary Information, Section E.) Moreover, to highlight the unique value of the SSSR-FPP method in the field of ultra-high-speed 3D imaging, we analyzed and compared the proposed method with three mainstream 3D reconstruction techniques [structure from motion (SfM)^48^, simultaneous localization and mapping (SLAM)^49^, multi-view stereo (MVS)^50,51^] in terms of imaging speed, number of point clouds, accuracy, independence from calibration, and cost-effectiveness. We further conducted a comparative experiment on the 3D reconstruction of a static plaster statue. The experimental results demonstrate that our method can achieve high-precision 3D reconstruction of 80244 point clouds at an imaging speed of 100,000 fps. Details are in the Supplementary Information, Section D, Figs. S10 and S11. Due to the artificial projection to help correlation matching and the ability of super-resolved imaging, our method has obvious advantages compared with other methods in terms of imaging speed, accuracy, and number of reconstructed point clouds. Therefore, it can be used to discover the physical mechanism behind transient phenomena and provide a powerful scientific tool for the study of transient events in aerospace, biomedicine, defense, and military fields.

### Further consideration about the SSSR-FPP method

Though the proposed method is capable of achieving single-frame super-resolution 3D reconstruction, it is essential to keep a clear mind and recognize that the SSSR-FPP method, despite its effectiveness, is not omnipotent. Several inherent limitations of deep learning methods should be taken into consideration carefully.

(1) The network basically obtains the mapping relationship between the desired image and the raw image through the learning of a huge amount of paired data and the adjustment of hyperparameter terms. From the opposite perspective, DNNs can be pretty fragile due to the impartial principle that information cannot be “formed from nothing”. In other words, if we do not employ a proper network model and training algorithm or fail to feed it with the correct type of data reflecting the real underlying physics, the neural network may yield poor performance.

(2) Deep learning approach eliminates the traditional approach’s reliance on handcrafted priors and adequately utilizes the high-frequency texture information “hidden” in the observed fringe pattern. More importantly, the reconstruction ability of computational imaging technology is greatly limited by “the accuracy of forwarding mathematical modeling” and “the reliability of reverse reconstruction algorithm”.

Recognizing these challenges, we implemented a physics-based procedure for the generation of training datasets instead of using simulations or direct down-sampling methods. Although adjusting the lens focal length to acquire samples at different image resolutions is indeed labor-intensive and imposes stringent requirements on the stability of the imaging system, these efforts are essential to overcome the “domain mismatch” problem. To alleviate these issues, we tried to explore optimization strategies for dataset generation. In the Supplementary Information, Section C and Fig. S7, we demonstrate the application of dataset generation techniques based on “digital twin” and transfer learning^52,53^ in our method, we show that the approach of combining virtual and real datasets can achieve a performance (MAE = 0.1037 rad) close to that of the approach with all real datasets (MAE = 0.0872 rad). The gap between these two approaches diminishes as the number of datasets gradually increases. The experimental results demonstrate the effectiveness of the dataset generation technique based on “digital twin” and transfer learning in reducing the dataset acquisition cost in the SSSR-FPP method. To address the issue of data imbalance in single-frame absolute phase retrieval and super-resolution, the raw low-resolution images fed into the network come from two cameras of different viewpoints, in addition to the physical model-based data acquisition approach, which inherently incorporates a specific parallax that facilitates removing phase ambiguity (the sub-pixel offset between two different views also contribute to the image super resolution). Finally, for highly reflective objects, the severely limited exposure time in ultra-high-speed imaging mode caused no overexposure in all cases of our experiments. However, establishing accurate datasets unaffected by overexposure is the most important challenge we face. To handle this issue, we combined a multi-exposure fusion algorithm^54^ in dataset production to eliminate the adverse effects of overexposure on achieving the wrapped phase (refer to Supplementary Information, Section C and Fig. S6). The results in Fig. S6 demonstrate that the introduction of the multi-exposure fusion algorithm generates composite optimal exposure fringe images, yielding global high-quality wrapped phase maps and effectively improving the 3D reconstruction accuracy of locally overexposed regions.

However, it is also important to acknowledge that our method has certain limitations. For instance, the diversity and complexity of samples that SSSR-FPP can effectively handle are still somewhat restricted. Furthermore, due to the inherent underdetermined nature of the pixel super-resolution problem, there also exists a fundamental limit to the degree of resolution improvement that can be achieved by deep learning. There are several intriguing avenues that merit further exploration to address these challenges. For example, incorporating a system point spread function (PSF)^55,56^ into our network design could potentially address this issue to a certain extent, or modeling the full-precision^57^ 3D geometry to explore the limits of accuracy. In addition, if we want to further extend the measurement volume, we not only need to customize the imaging focal length and field of view of the projector and imaging camera but also need to take into account the light power of the projector.

### Conclusions

In this work, we have reported SSSR-FPP, a learning-based ultrafast 3D imaging technology that uses only one pair of low-SNR, low-resolution, and pixelated fringe patterns to achieve high-resolution absolute 3D shape measurement of dynamic events. To our knowledge, this is the first demonstration of ultra-high-speed FPP 3D imaging at an unprecedented speed of 100 kHz. SSSR-FPP exploits the significant speed gain achieved by reducing the imaging window of conventional high-speed cameras while “regenerating” the lost spatial resolution with deep learning, resulting in a boost in 3D frame rate up to more than one order of magnitude without compromising spatial resolution. Moreover, owing to its single-shot nature, the SSSR-FPP method fundamentally overcomes the phase-shifting errors and associated artifacts induced by object motion. Finally, by simply utilizing imaging sensors with a higher frame rate in conjunction with a high-power light source and large-aperture imaging optics could, in principle, further push 3D imaging frame rates up to the million-frame-per-second regime. Experimental results suggest that SSSR-FPP is expected to offer new insights for studying a multitude of ultra-fast dynamic processes, advancing our knowledge across various scientific disciplines.

## Materials and methods

### Optical setup

The SSSR-FPP prototype is shown in Fig. 1, which is composed of two high-speed scientific cameras (Vision Research Phantom V611) and a customized DLP projection system with an XGA resolution (1024 × 768) DMD chip. A 24 mm-85 mm lens (Nikon AF-S, an aperture is continuously adjustable from f/3.5 to f/4.5) was mounted on the scientific camera, and the aperture (F-number) of the lens was fully open to permit the maximum light flux for imaging. In experiments, the cameras operated within a localized readout window (160 × 160) with a focal length of 24 mm, so that it is capable of capturing consecutive images at a frame rate of 100,000 fps with a maximum exposure time of 9.5 *μ*s. Besides, to generate training datasets, the cameras were set to the resolution of 480 × 480 when capturing high-resolution fringe images, with a focal length of 72 mm, a lower frame rate (2,2000 fps), and a longer exposure time (45 *μ*s). By omitting any grayscale features and displaying binary images on the DMD, the system refresh rate was driven at 2,2000 fps, which isis precisely synchronized by a custom-built FPGA circuit. For more details, see Supplementary Information, Section B about the optical system setup and hardware synchronization.

### Methodology overview

The first workflow of the deep learning-based SSSR-FPP framework involves generating training datasets. Instead of creating datasets through simulations (e.g., based on simple pixel merging or optical transfer function modeling), in the proposed solution, the desired 3 × super-resolved results corresponding to the low-SNR, low-resolution fringe images were obtained experimentally based on the same experimental setup but tripling the focal length of the lens (from 24 mm to 72 mm), as demonstrated in Fig. 1. This approach allows the SSSR-FPP framework to learn more potential information reliably from experimental data with “physical meaningful” prior knowledge about the image formation process, without requiring additional computational resources. Further details on the dataset preparation for network training are provided in Supplementary Information, Section C.

Then, the workflow of the SSSR-FPP framework comes to train the neural networks. SSSR-FPP employs two collaborative but functionally different CNNs, CNN1 and CNN2, which were trained independently and sequentially. CNN1, which combines a dual regression architecture and a composite loss based on physics and data, accepted as input a low-resolution (160 × 160) and low-SNR fringe image. Corresponding labels were 3 × super-resolved (480 × 480) numerator and denominator terms of the wrapped phase function, which could be calculated to achieve the high-resolution wrapped phase. The task is to perform super-resolved phase retrieval via deep learning. For CNN2, the training process established a mapping from an ambiguous wrapped phase to an absolute phase map. CNN2 is a framework embedded with geometric constraints and phase unwrapping, and its input was a pair of low-resolution fringe images, super-resolved wrapped phase maps (predicted by CNN1), and the reference plane information. The label for CNN2 was the absolute phase map, obtained by the multi-frequency phase shifting method (12-step phase-shifting and 3-frequency temporal phase unwrapping with frequencies of 1, 8, and 32). Refer to Supplementary Information, Section A for more network training details.

During the implementation stage, i.e., in the transient experiments, CNN1 received a low-resolution fringe image and output super-resolved numerator and denominator terms of the wrapped phase. The architecture of CNN1 incorporates the physical model of the phase-shifting method, bypassing the difficulty of predicting 2*π* jumps in the wrapped phase function. The dual regression architecture and composite loss enable CNN1 to be driven by both experimental data and physical models. Then, the high-resolution wrapped phase, along with reference plane information, was fed into CNN2, which deciphered an unambiguous absolute phase map. Although the output absolute phase output is relatively “coarse” due to factors such as environmental light, large surface reflectivity, and discontinuities, it is sufficient to resolve the precise fringe order of the wrapped phase. This allows the generation of a high-resolution and high-precision absolute phase. Finally, with the pre-calibrated parameters, super-resolved 3D imaging at 10,0000 fps can be realized.

### Image acquisition and 3D reconstruction

In image acquisition, the focal length of the imaging lens needed to be adjusted repeatedly during data collection. To automate the whole process, an electro-mechanical lens mount adapter was employed for electronic control of focus and aperture. Moreover, a phase-correlation-based image registration algorithm was implemented to align the LR-HR image pair precisely for dataset preparation. In this work, the training dataset consists of 1000 groups of fringe images with corresponding ground truth data (for additional information about the optical setup and training data acquisition details, refer to Sections B and C of the Supplementary Information). It also should be noted that when the relative position of the camera or projector is changed the established mapping function of the training data will be destroyed and the process needs to be restarted completely. To maintain the model’s generalization capabilities and ensure its accuracy, retraining is recommended when alterations occur to the imaging setup or environmental conditions. This process necessitates the acquisition of new HR and LR image pairs. These image pairs serve as the foundation for enhancing or retraining the existing network, thereby ensuring that the training model maintains high performance and adaptability in diverse real-world applications.

We designed two CNNs (CNN1 and CNN2) with identical architectures but distinct input and output configurations. Two CNNs (CNN1 and CNN2) perform the specific functions of super-resolved phase retrieval and phase unwrapping, respectively. CNN1 and CNN2 operate independently on their respective tasks and strive to reach an optimal state. After learning from the massive dataset, the properly trained model can “regenerate” the lost spatial resolution and predict high-accuracy absolute phase information from only one pair of low-quality pixelated fringe images, enabling single-shot, super-fast and unambiguous 3D surface imaging. (the corresponding system acquisition process and reconstruction workflow are detailed in Supplementary Visualization 1).

## Supplementary information

Supplemental material for single-shot super-resolved fringe projection profilometry (SSSR-FPP): 100,000 frames-per-second 3D imaging with deep learning

Visualization 1

Visualization 2

Visualization 3

Visualization 4

Visualization 5

Visualization 6

Visualization 7

Visualization 8

## Data Availability

All data are available in the main text or the supplementary materials from the corresponding author upon reasonable request.
