# Supplementary material for: Single-shot super-resolved fringe projection profilometry (SSSR-FPP): 100,000 frames-per-second 3D imaging with deep learning
Source: Light Sci Appl. 2025 Feb 7;14:70. doi: 10.1038/s41377-024-01721-w (PMC11802878; doi:10.1038/s41377-024-01721-w)
Supplement: Supplementary file 1 — Supplemental material for single-shot super-resolved fringe projection profilometry (SSSR-FPP): 100,000 frames-per-second 3D imaging with deep learning [file 41377_2024_1721_MOESM1_ESM.pdf]

# Supplementary Information:

# Single-shot super-resolved fringe projection profilometry (SSSR-FPP): 100,000 frames-per-second 3D imaging with deep learning

**Bowen Wang[1,2,†], Wenwu Chen[1,2,†], Jiaming Qian[1,2], Shijie Feng[1,2,***], Qian Chen[1,2,**], and Chao Zuo[1,2,*]**

[1]Jiangsu Key Laboratory of Spectral Imaging and Intelligent Sense, Nanjing University of Science and Technology, Nanjing, Jiangsu Province, China.
[2]Smart Computational Imaging (SCI) Laboratory, Nanjing University of Science and Technology, Nanjing, Jiangsu Province 210094, China.
[*]zuochao@njust.edu.cn
[**]chenqian@njust.edu.cn
[***]shijiefeng@njust.edu.cn
[†]these authors contributed equally to this work

## ABSTRACT

This document provides supplementary information for “Single-shot super-resolved fringe projection profilometry (SSSR-FPP): 100,000 frames-per-second 3D imaging with deep learning”. We present a deep learning-based ultrafast 3D imaging method, termed single-shot super-resolved FPP (SSSR-FPP), that enables 3D imaging at 100,000 Hz.

## Contents:

**A. Network Design and implementation**
**B. Optical system setup and hardware synchronization**
**C. Dataset preparation for network training**
**D. Analysis of spatial resolution and comparison with other methods**
**E. Analysis and comparison of different fringe pattern schemes**
**F. Measurement results of diverse static plaster models**
**G. Dynamic 3D measurement results in multiple scenarios**

## A. Network Design and implementation

### Network architecture design

The deep learning technique breaks the reliance of traditional methods on prior knowledge and efficiently utilizes the raw information "hidden" in the original fringe image. More importantly, the reconstruction ability of computational imaging technology[1] is greatly limited by "the accuracy of forwarding mathematical modeling" and "the reliability of reverse reconstruction algorithm." Due to the progressively requirement to achieve a single 3D reconstruction, considerable attention has been paid to alleviating under-determined problems (or ill-posed inverse problems[2]) that exceed the Nyquist criteria[3]. Recovering the under-determined information is analogous to the processes of computer vision and computational imaging, presenting an inverse solving problem that often lacks optimality in terms of solution existence, uniqueness, and stability. More precisely, a sufficient number of projected images are required, thereby performing a stable solution (the measurement data needs to satisfy the basic principle of solving unknown pixel values). It is possible to minimize $f$ by mapping a massive data samples (deep learning approach gradually reduces the loss function through multiple epochs and updates the weight parameters through feedback), and thus $Y$ can be precisely learned [Fig. S1(b)]. In particular, U-net[4] has demonstrated remarkable success in addressing the mapping functions for various ill-posed medical imaging problems, thus confirming the potential of utilizing neural networks to constrain inverse problems of super-resolved phase retrieval and phase unwrapping.

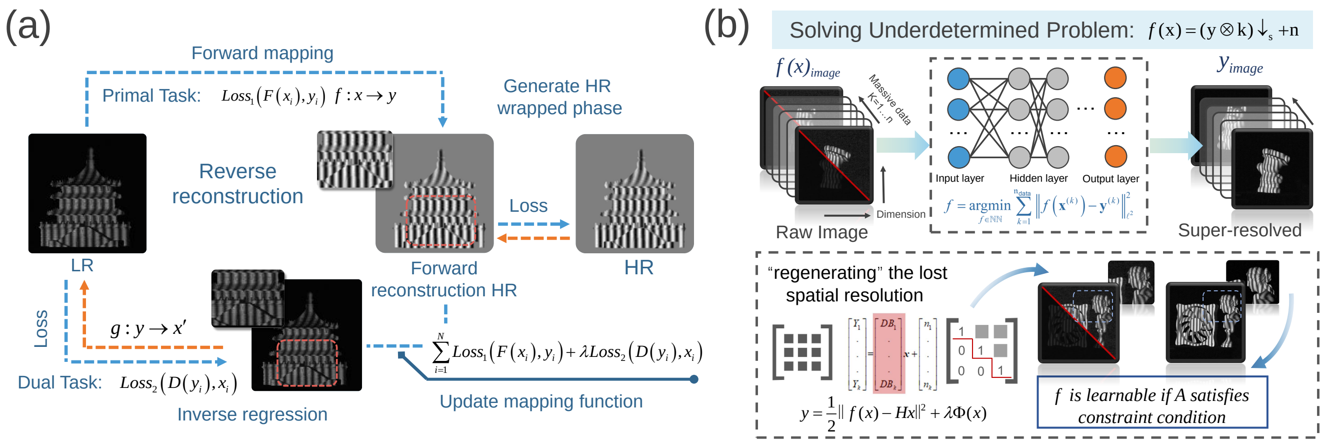


**Figure S1.** (a) A dual regression architecture in the SR structure. (b) Description of solvability of underdetermined inverse problem $AY = F(x)$.

In this section, we further provide an analysis of the selection strategy and the core architecture of the proposed network. For the network architecture selection, we apply the encoding-decoding structure to achieve phase retrieval[5] and phase unwrapping, respectively, as shown in Fig. S2(a). We construct two convolutional neural networks (CNN1 and CNN2) with the same structure (except for different inputs and outputs) to learn to obtain the high-quality phase information and unwrap the wrapped phase. Since one of the inputs of CNN2 is the output of CNN1, in our workflow, CNN1 was trained first, and then

CNN2 was trained with the predicted unwrapped phase map along with the reference images (a pairs of fringe images and reference plane information). Driven by extensive training datasets, the neural network can gradually "learn" to transfer one high-frequency fringe image into the "physically meaningful" and "most likely" absolute phase, instead of "step by step" as in conventional approaches. Both networks are trained separately and converged to an optimal state. Instead of directly adopting an end-to-end learning scheme linking the input fringe image to the output phase map, we choose to predict the numerator and denominator terms of the wrapped phase map from an input fringe image. Network learning performs feature extraction when the input passes through the coding layer. Compared with the maximum pooling layer, the convolution layer with a step size of two can retain the feature information to the greatest extent. In addition, we deleted the batch normalization (BN) process after the convolution layer and the activation function. It is mainly considered that the normalization operation is vulnerable to disturbances in the feature information learned by the convolution layer, which is contrary to our purpose.

Due to the inherent depth ambiguity in FPP, a single fringe pattern is typically insufficient to uniquely determine the fringe order, particularly when dealing with isolated surfaces or surface discontinuities[6]. The absence of supplementary auxiliary data can lead to a simple input-output network structure, which directly links the fringe image to the absolute phase, yielding a vulnerable estimation. This is especially evident when the measured surface features sharp edges, discontinuities, or significant variations in reflectivity[7]. Based on this consideration, our deep neural network is trained to predict the intermediate numerator and denominator of the arctan function for unwrapped phase maps. The proposed method circumvents the complexities associated with the abrupt $2\pi$ phase wraps, thereby enhancing the quality of the phase estimate. Additionally, the resemblance between the input and output images bolsters the neural network's predictive capabilities. Meanwhile, for the absolute phase solution, we leverage information from dual camera perspectives and incorporate a priori reference plane data. By applying joint constraints, we can further ensure the generation of high-fidelity absolute phase information.

At present, the mainstream network structure models tend to develop in a deeper direction[8]. A deeper network model implies better nonlinear representability, which also means that it is capable of learning more complex transformations and adapting to perform more complex feature inputs. However, the information extracted by the middle layers is not fully utilized, which is a common accompaniment problem, reducing the network's super-resolution ability. The inclusion of skip connections in the residual structure is beneficial for improving gradient propagation and mitigating the issue of vanishing gradients associated with deeper networks, and thus the network can center on more informative features between each dimension. By employing this approach, a comprehensive range of image information at various scales is retrieved and effectively utilized in conjunction with one another.

An example of CNN2 is introduced to illustrate the internal structure of the constructed network, as shown in Fig. S2(b). The network takes a 3D tensor of size $(H,W,C_0)$ as input, where $(H,W)$ represents the dimensions of the input images and $C_0$ denotes the number of input channels, which is five in this particular case. Each convolutional layer utilizes a $3\times3$ kernel size, resulting in a 3D tensor output of shape $(H,W,C)$, where C = 128 denotes the quantity of filters employed in each convolutional layer.

- In the first path of CNN1, the network adopts an encoder-decoder structure. Convolution layers,

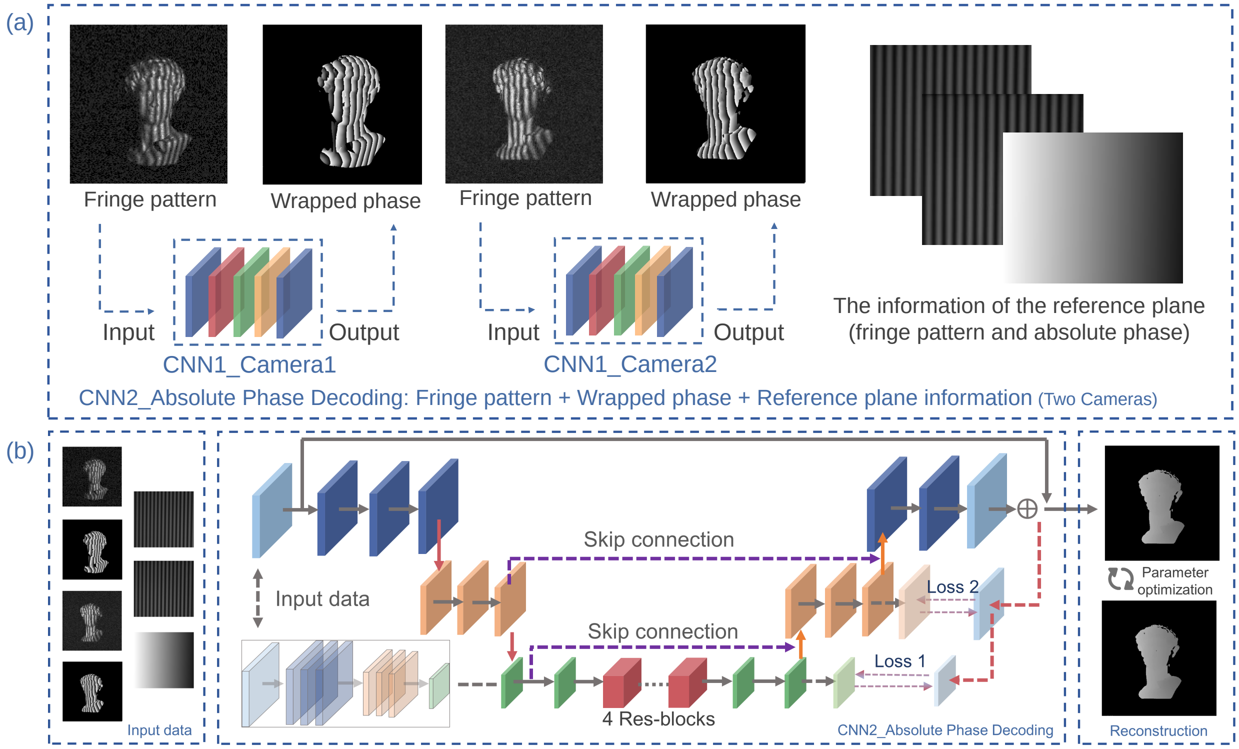


**Figure S2.** Overview of the proposed network's selection strategy and core architecture. (a) Details of the implementation for the distinct network structures and the inputs required for CNN2. (b) The architectural layout of CNN2.

followed by a group of residual blocks, are applied as feature extractors to provide texture features from raw images. Convolution with a stride of two is devoted to diminishing the dimension of feature images. Deconvolution layers can up-sample feature maps and recover the high-frequency information. Rectified Linear Unit (ReLU) layers are inserted following each convolution layer and deconvolution layer. Compared with the max-pooling layer, the convolution layer with a step size of two can retain the feature information to the greatest extent. Skip connections play the role of transmitting image feature information while also alleviating the problem of gradient disappearance. We implemented a nonlinear fit by adding a residual module to transmit channel feature information, which facilitates the reconstruction of HR images.

- The existing methods only focus on the forward mapping process from the LR image to the HR image. Nevertheless, the potential mapping space of the under-determined function is tremendously challenging in the training process. In order to address the existing problem, we posed a dual regression term in the SR structure, as presented in Fig. S1(a). Considering the asymmetric mapping of learning, an additional constraint is deployed, which can be regarded to mutually retain the dual-regression mapping between HR and LR images to improve the network robustness and the generalization capability under real-world settings. In addition, predicting the wrapped phase from the arctangent function bypasses the difficulties associated with reproducing abrupt $2\pi$ phase wraps,

and thus, obtains a high-quality phase estimate.

## Network training

The network is configured with a batch size of 4 and trained for 200 epochs. Empirically, an adaptive moment estimation (ADAM) optimizer is utilized to optimize the network structure, with an initial learning rate set to $10^{-4}$. The model training is conducted on an Intel Core $^{TM}$ i7-9700K CPU @ 3.60GHz$\times$8 and RTX 3090Ti graphics card platform, using Pytorch 1.3.0 under Ubuntu 16.04 operating system.

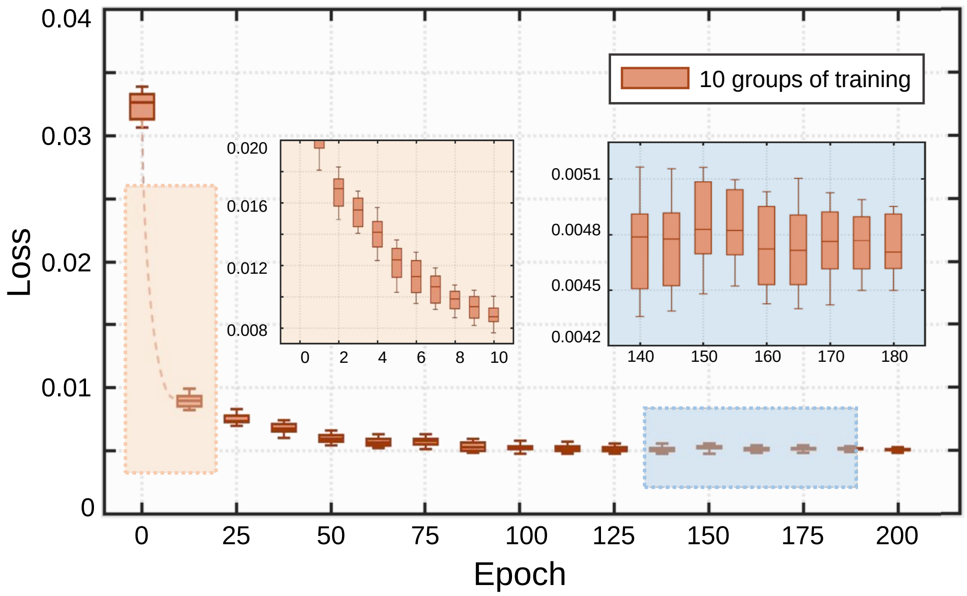


**Figure S3.** Stability analysis of loss curve of ten dissimilar training sets.

In order to yield reliable predictions, the network model simultaneously constrains the forward generation process and reverse regression process, with dual loss functions balancing each other to produce the overall loss function. The mixing loss is measured at each epoch, which is fed back through the optimizer to update the network parameters. By constraining the minimization loss function, the network implements an accurate reconstruction of the input data during the training period, emphasizing the valuable information and suppressing the irrelevant information. To perform network training, the information similarity between the predicted and input image pairs needs to be accurately evaluated to minimize the information loss and thus effectively retain texture detail information. The loss function of the proposed model is defined as:

$$Loss = \sum_{i=1}^{N} \mathrm{Loss}_1 \left(F\left(x_i\right), y_i\right) + \lambda \, \mathrm{Loss}_2 \left(D\left(y_i\right), x_i\right) \tag{S1}$$

where $x_i$ and $y_i$ represent the input LR and output HR images, respectively. $Loss_1\left(F\left(x_i\right), y_i\right)$ and $Loss_2\left(D\left(y_i\right), x_i\right)$ describe the loss functions of forward regression and inverse regression tasks, respectively. The reconstructed image $F(x_i)$ is constantly approaching the similarity with its corresponding HR image

in the training process. The similarity between the predicted image $D(y_i)$ and the forward input LR image gradually converges during the regression process. In this case, we assign a weight distribution of 0.1 to $\lambda$ for the hybrid loss function.

If $F(x_i)$ represents the high-resolution image, then the image $D(y_i)$ in the inverse regression model should closely resemble the low-resolution image. Under such constraint, it motivates the network to achieve a more realistic approximation of the predicted data and thus achieving robust image reconstruction. Taking CNN1 as an example, the loss function curve of training is shown in Fig. S3. We also carried out relevant stability experiments and scrambled the training data set to obtain the stability test results undergoing disordered data.

## B. Optical system setup and hardware synchronization

The SSSR-FPP principle prototype is composed of two high-speed scientific cameras (Vision Research Phantom V611) and a customized DLP projection system with an XGA resolution ($1024 \times 768$) DMD (The corresponding 3D movie is provided in Supplementary Video 1). We drive the DMD at an ultra-fast refresh rate by omitting any grayscale capabilities and setting the fixed mode of active time and blanking time to 45.45 µs and 0 µs, respectively, to guarantee the refresh rate of the single image. The light source is a built-in green LED module with a brightness of 600 lm. The specific timing control is depicted in Fig. S4(b). The system employs a high-speed camera with a frame rate of 100,000 fps, a maximum image resolution of $160 \times 160$, and an exposure time of 9.5 µs. A 24 mm - 85 mm (in the experiment, the focal lengths were set to 24 mm and 72 mm, respectively) lens (Nikon AF-S, the aperture is continuously adjustable from f/3.5 to f/4.5) was mounted on the scientific camera. The aperture (F-number) of the imaging lens is fully open to permit the maximum light flux for imaging. The specific trigger control signal is shown in Fig. S4(a) (high precision triggering to ensure signal synchronization through custom development boards). The traditional DFP technique commonly utilizes 8-bit sinusoidal fringe patterns and is constrained by the maximum refresh rate of the projector, typically set at 120 Hz, limiting its measurement speed. While acquiring multi-phase-shifted fringe images, the presence of target movement poses a challenge for traditional DFP measurement techniques. As a solution, binary defocusing techniques have been investigated to address this limitation by producing quasi-sinusoidal fringes with binary patterns through defocusing of the projector lens. Stable switching between HR and LR images is realized through electric zoom and focus, as illustrated in Fig. S4(c).

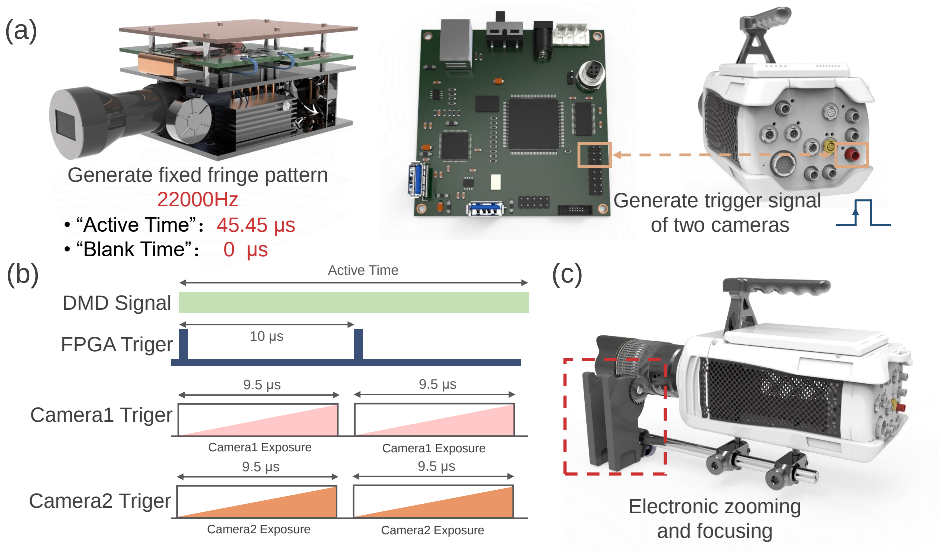


**Figure S4.** Schematic diagram of signal synchronization trigger. (a) Schematic diagram of the SSSR-FPP system developed. (b) Timing diagrams of trigger settings applied under the experiment. (c) Stable switching between HR and LR images is realized by electric zoom and focusing.

## C. Dataset preparation for network training

In recent years, deep learning has emerged as a powerful tool for solving problems through data-driven learning, which is primarily driven by the availability of massive datasets. Especially for the under-determined image restoration problem of single-frame image super-resolution. In most of the dataset construction methods, LR sequences are synthesized from HR sequences using simple degradation models, e.g., bicubic down-sampling or direct down-sampling with Gaussian smoothing. It is evident that these simplistic degradation models are inadequate for accurately representing the intricate degradation process of SR in real-world scenarios. Even if these datasets can serve as a reasonable baseline for investigating and evaluating SR algorithms, the traditional degradation model for LR-HR pair generation makes it challenging in practical application due to the complex degradation process of the real world. Based on the datasets constructed by such methods, the super-resolution predicted images tend to be too smooth and prone to visual artifacts (not robust to real reconstructed data). This prompted us to rekindle the interest in building a real-world SR dataset to bridge the gap between synthetic and real. With the above analysis, we built a real-world super resolution dataset to capture pairs of LR and HR images of the same scene by adjusting the focal length of the camera lens. Our training dataset contains multiple types of objects so as to facilitate the versatility of the network in performing different scenarios. We trust that building the dataset through such realistic models is the most conducive to mapping the network model compared to simulation methods. As we had forecasted, such image acquisition methods are vulnerable to minor displacement errors between image pairs. To address such problems, we develop image registration and alignment algorithms to progressively align image pairs with different resolutions. The constructed dataset has been shown through experimental demonstration to effectively tackle the real-world single image super-resolution problem, delivering better performance.

In general, imaging lenses with longer focal lengths could capture finer details with a scaling factor equal to the ratio of focal lengths. Considering the severe distortion of wide-angle lenses and the poor quality of cropped images, cameras equipped with 24 mm-equivalent lens and 72 mm-equivalent lens are employed to construct the dataset in the proposed system. The distance from the measured object to our system is 1.5 m [Fig. S5(b)]. In order to obtain precise parameter estimates, the multi-step phase-shifting algorithm is employed to produce the ground truth data for our neural networks. We prefer to form the phase-shifting method with more steps, such as 12, to obtain higher-quality phase-related information. The sequence captured by the camera with a 72 mm-equivalent lens is considered as the ground truth HR sequence for captured image pairs, while the sequence captured by the camera with a 24 mm-equivalent lens is adjusted to produce the corresponding LR sequence [Fig. S5(a)], generating a dataset for $\times 3$ SR. The acquisition process of corresponding data sets is also shown in Supplementary Video 1. It is worth mentioning that a triple scaling is currently the main requirement for super-resolution tasks (a more significant multiplier boost would, in principle, require a more massive amount of data to support it). In contrast to existing synthetic SR datasets, our real SR dataset is collected in a real-world setting, which naturally takes into account the complex degradation factors in real scenarios. The proposed registration and rectification algorithms are designed to align multiple images of a single object with a reference image and to correct the distortion caused by lens aberration.

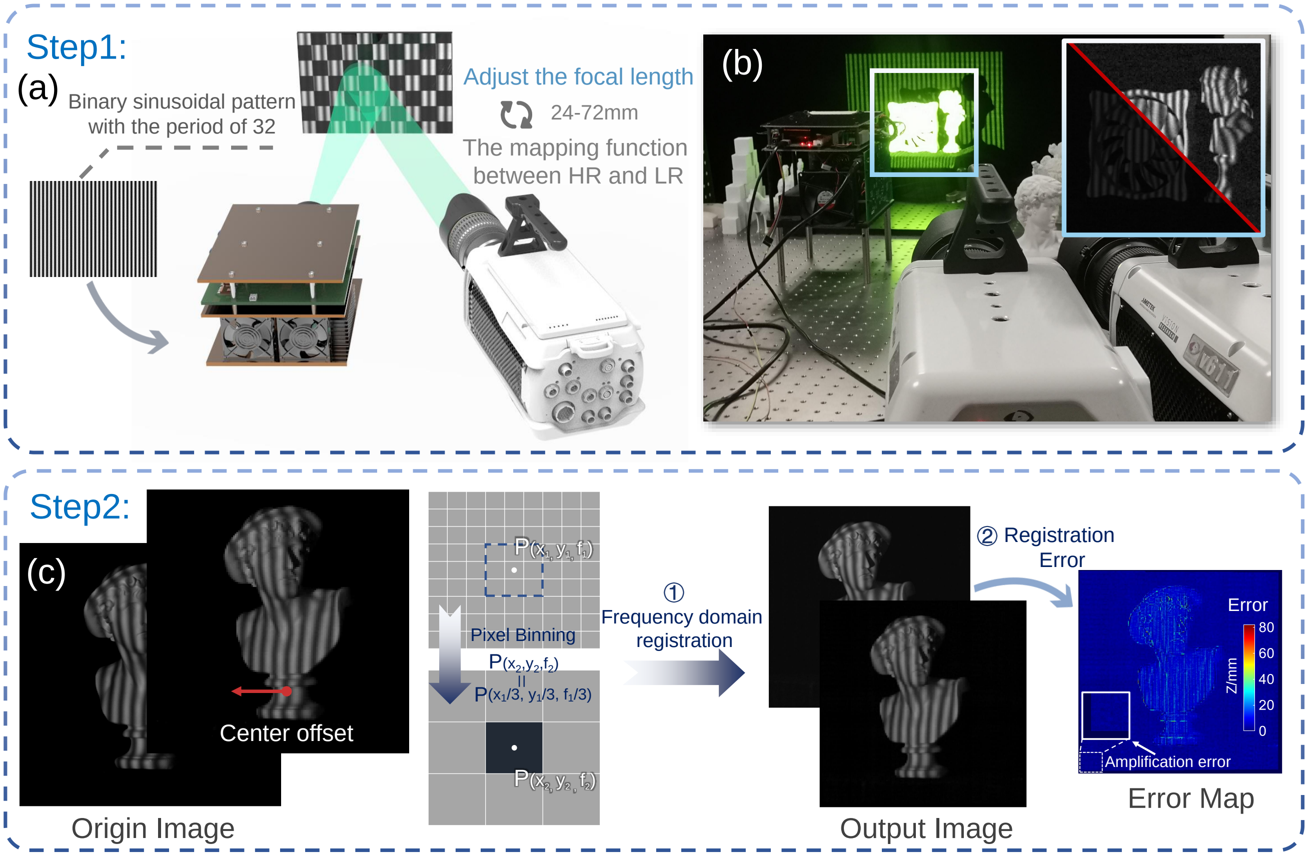


**Figure S5.** Dataset preparation for network training. (a) Experimental calibration setup. (b) Experimental setup with zoomed-in detail. (c) Frequency domain registration process.

The selected lens is designed for full-frame photography and is frequently employed with partial readout in the central field of view during high-speed measurement scenarios. Consequently, the captured images are typically under the paraxial approximation, which significantly reduces distortion errors, particularly with shorter focal length lenses. Upon capturing the absolute phase, our imaging system undergoes a detailed calibration process. In this paper, we implement Zhang's calibration method[9] to establish preliminary distortion parameters. By employing a calibration target, we accurately determine the camera's intrinsic parameters and distortion coefficients, subsequently correcting image distortions to ensure the precision and reliability of the outcomes. Furthermore, our dataset is collected under consistent conditions of field of view and focal length. Through comprehensive dataset mapping, the network inherently comprehends the correlation between high and low resolution image pairs. The comprehensive learning process encompasses the acquisition of distortion parameters, enabling the system to adeptly handle variations in image quality and focus. However, there were still minor misalignments and differences in luminance between the LR-HR sequences. Figure S5(c) illustrates an instance where a slight variance in global luminance and color between the LR and HR frames is noticeable, due to variations in viewing angle and response rate between two cameras. We addressed the corresponding issues through exposure compensation and the development of alignment algorithms. Undoubtedly, such methods also

encounter challenges in practical imaging scenarios, e.g., the forward model remains an approximation of reality and has inherent defects of the lens (the optical center is also shifted when zooming on the focal length).

In this paper, we develop an image registration algorithm to align image pairs to progressively build our dataset, which is divided into coarse registration and fine registration.

- Geometric transformation of image rotation, translation, and deformation is achieved by searching the corresponding image feature points, eliminating the deformation and displacement errors between distinct lenses as much as possible. When the distortion error between the two images is eliminated (the error caused by projection transformation under different focal lengths can be corrected by finding feature points), we deem that the registration error between the two images is only the displacement between pixel levels.

- For the adjacent sub-pixel image alignment, we adopt the frequency domain cross-correlation method for registration, realizing the error correction between each image. It is worth noting that we employ the electric limit switch to continuously zoom and focus the imaging lens. Therefore, in principle, we only need to perform accurate registration once to complete the registration of all data sets.

In order to obtain precise parameter estimates, we employ the multi-step phase-shifting algorithm to calculate the ground truth data for our neural networks. The formation of the multi-step phase-shifting fringe patterns is as follows:

$$I_n^p(x^p, y^p) = a + b\cos\left(2\pi f x^p - \frac{2\pi n}{N}\right) \tag{S2}$$

where $(x^p, y^p)$ represents the pixel coordinate of the projector, and index $n = 0, 1, 2, ..., N-1$ ($N$ is the number of phase-shifting steps). Parameters $a$, $b$, $f$ are the mean value, amplitude and spatial frequency, respectively. In our experimental setup, we fixed the values of $a$ and $b$ at 127.5 (corresponding to the fringe pattern projected onto the object) and set $f = 32$. The projector was used to display the generated multi-step phase-shifting fringe patterns onto various objects under measurement. Subsequently, multiple sets of phase-shifting fringe patterns were recorded for each scene. The acquired phase-shifting images can be expressed as:

$$I_n(x,y) = A(x,y) + B(x,y)\cos[\varphi(x,y) + 2\pi n/N] \tag{S3}$$

where $I_n$ refers to the $(n+1)$th captured image, $n = 0, 1, ..., N-1$, $(x, y)$ is the camera pixel coordinate. $A$ represents the average intensity map, $B$ denotes the fringe amplitude map, $\varphi$ stands for the phase, and $2\pi n/N$ represents the phase shift. Through the standard N-step phase-shifting algorithm, we can calculate the corresponding ground truth data with the least square method:

$$\varphi(x,y) = \arctan\frac{M(x,y)}{D(x,y)} = \arctan\frac{\sum_{n=0}^{N-1} I_n(x,y)\sin(2\pi n/N)}{\sum_{n=0}^{N-1} I_n(x,y)\cos(2\pi n/N)} \tag{S4}$$

With the aid of Eq. S4, the molecular term $M$ and denominator term $D$ of the arctangent function can be yielded. It is worth noting that we recommend using the phase-shifting method with a higher number of steps, such as the 12-step phase-shifting method, to obtain higher-quality phase information.

During the period of training dataset preparation, we photographed a variety of objects made of different materials (plastic, plaster, metal, ceramic, etc.) to generate diverse datasets. This allows the network to see as many different scenes and conditions as possible, thereby improving the network's ability to generalize to various applications. However, when capturing LR and HR image pairs of different materials, we may encounter the problem of localized overexposure, particularly in measuring highly reflective objects like metal. Recall that when capturing an LR image ($160 \times 160$), the camera works at a frame rate of 100,000 fps with an exposure time of 9.5 µs. This severely limited exposure time does not result in localized overexposed areas on highly reflective objects, as shown in Fig. S6(a). However,

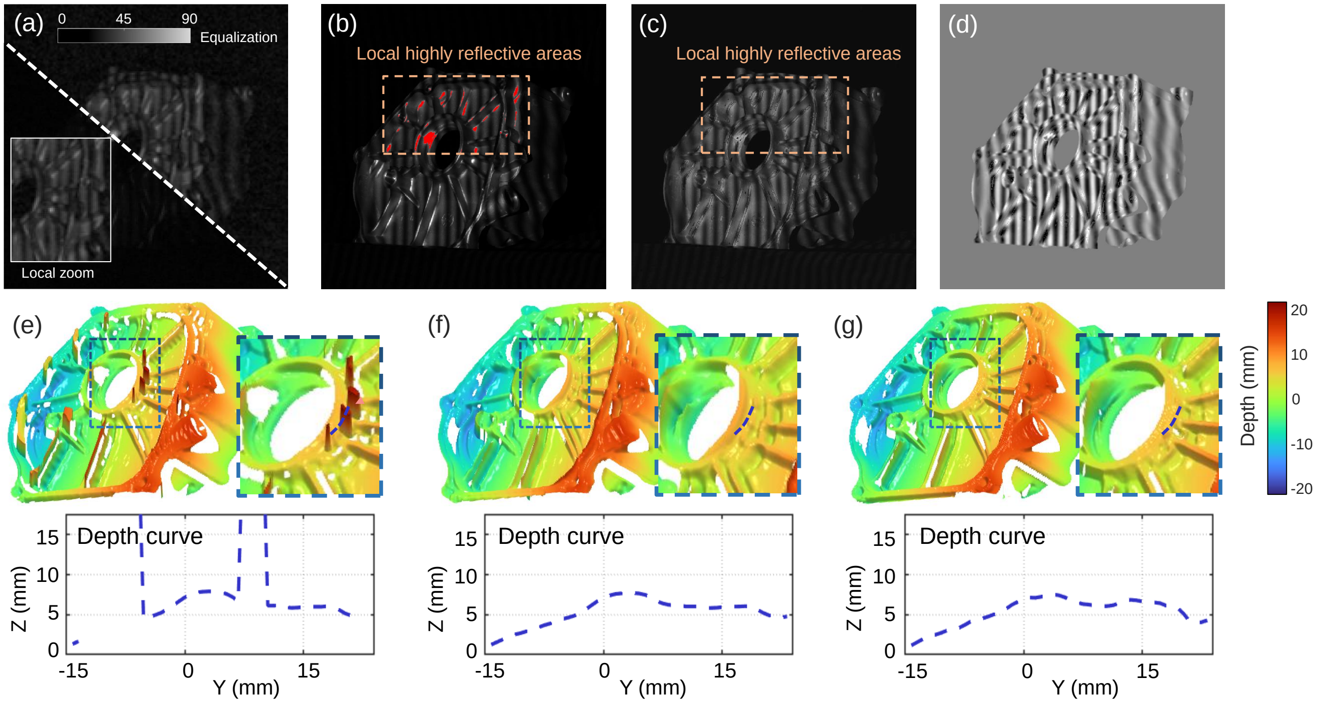


**Figure S6.** Reconstruction results of industrial parts. (a) Low-resolution fringe image (exposure time of 9.5 µs, no local overexposed areas). (b) High-resolution image (exposure time of 45 µs, presence of local overexposed areas, red area in the dashed box shows the overexposed regions). (c) Composite exposure fringe images obtained by the multi-exposure fusion algorithm (multiple exposure time of 5, 25, 45 µs, the area of the overexposed region is significantly reduced). (d) The denominator term of global high-quality wrapped phase map from (c). (e) 3D topography reconstructed by our method without the multi-exposure fusion algorithm in the dataset. (f) 3D topography after using the multi-exposure fusion algorithm in the dataset. (g) Ground truth (obtained by 12-step phase-shifting method and 3-frequency temporal phase unwrapping). Profiles (y-z) of 3D reconstruction results corresponding to the blue dot line in the magnified zoom region are also shown in (e), (f), and (g).

when the corresponding HR image ($480 \times 480$) is captured, at which time the frame rate is 22,000 fps and the exposure time is 45 μs, multiple areas of significant overexposure have occurred, causing significant distortion of phase information. In detail, the gray values of the overexposed regions in the HR image are oversaturated [as in Fig. S6(b)], which will degrade the wrapped phase values of the corresponding regions obtained from the multi-step phase shifting, thus affecting the final 3D reconstruction accuracy. Figure S6(e) shows the result of 3D reconstruction using the overexposed image directly, and it can be seen that the corresponding depth values are severely shifted. To reduce the degradation of imaging quality by locally overexposed regions, we combined a multi-exposure fusion algorithm[10] in dataset production to eliminate the influence of such unfavorable factors on achieving the wrapped phase. The multi-exposure fusion algorithm predicts appropriate exposure times for highly reflective objects based on the deduced statistics distribution of the reflectivity and generates composite exposure fringe image by selecting the pixels with optimal fringe quality from the raw fringes captured at the predicted multiple exposure times (in this work the exposure time is selected at 5, 25, 45 μs). The composite exposure fringe image is shown in Fig. S6(c). It can be seen that the area of the overexposed region is significantly reduced. The multi-exposure fusion algorithm can obtain a global high-quality wrapped phase map [the denominator term is shown in Fig. S6(d)] without the interference of overexposed regions. Figures S6(e) and (f) show the 3D topography of the industrial parts reconstructed by the proposed method before and after using the multi-exposure fusion algorithm in the dataset, respectively. Figure S6(g) shows the ground truth of 3D results. It can be noticed that using the multi-exposure fusion algorithm can effectively solve the effect of local overexposed regions on the 3D reconstruction results.

In addition, although the physics-based dataset generation method mentioned above can effectively alleviate the domain mismatch problem, there is no doubt that this method of generating datasets by repeatedly adjusting the lens focal length to acquire samples at different image resolutions is indeed labor-intensive and also imposes stringent requirements on the stability of the imaging system. Meanwhile, the phase-correlation-based image registration algorithm used to align LR-HR image pairs consumes additional computational resources, and when the number of datasets increases, the consumption of such resources will also increase proportionally. To alleviate these issues, we further explored a dataset generation method based on the idea of ”digital twin” and transfer learning[11,12]. In this method, we used Blender (a three-dimensional computer image software for creating and rendering models and visual effects) to establish a digital SSSR-FPP system that is twinned with the real system and generated simulated fringe images for making virtual datasets. The established virtual system and the produced virtual dataset are shown in Fig. S7(a). Meanwhile, we used the real system and the physics-based dataset generation method to generate real datasets [also shown in Fig. S7(a)]. In the network training stage, we first used the virtual dataset to pre-train the network and then used the real dataset combined with the transfer learning to train the network for the second time. The introduction of virtual datasets can reduce the dependence of network training on real datasets to a certain extent. Recall that in the SSSR-FPP method, the acquisition of real datasets is time-consuming. Therefore, introducing the dataset generation technique of digital twin and transfer learning into our method can reduce the cost of dataset acquisition.

We conducted a comparative experiment to verify the effectiveness of combining virtual and real

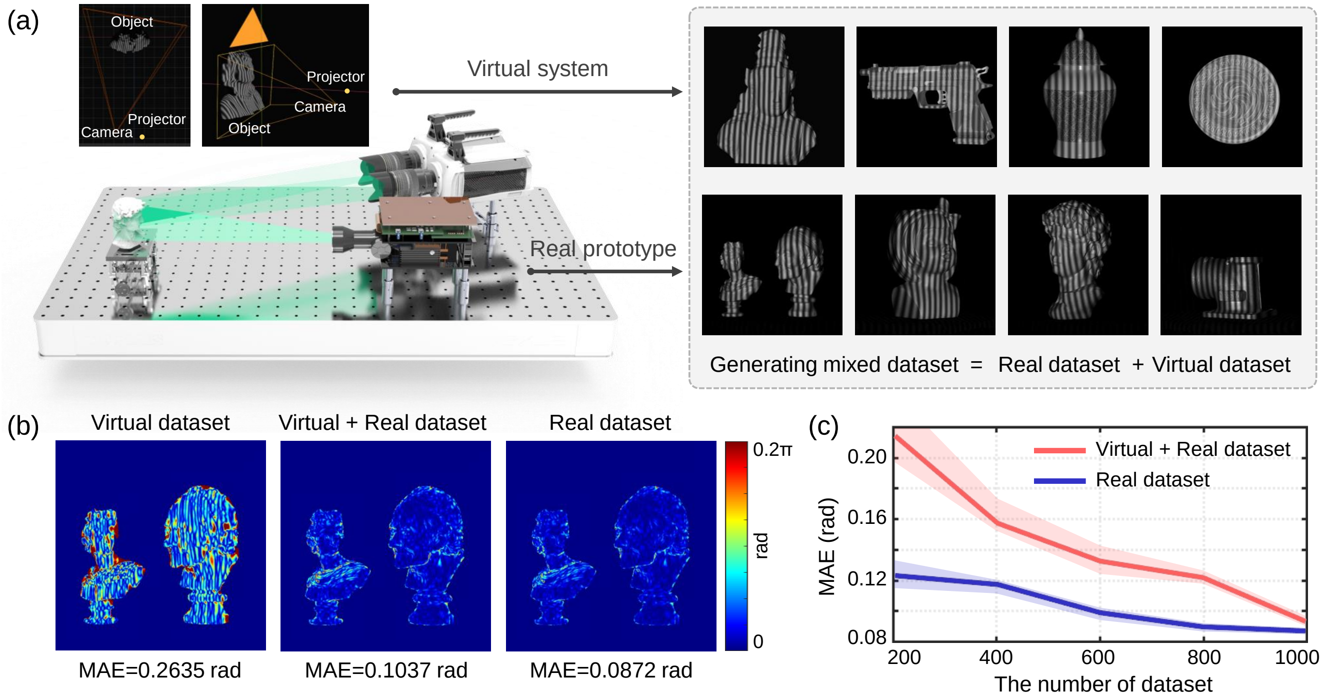


**Figure S7.** A comparative experiment to verify the effectiveness of combining virtual and real datasets. (a) The virtual system in Blender with the produced virtual dataset, and the real system with the produced real dataset. (b) The MAE of the reconstructed absolute phase by the network trained by virtual datasets, virtual + real datasets (pre-training and transfer learning), and real datasets, respectively. (The number of datasets = 1,000) (c) The impact of the number of datasets on the experimental results. The blue line represents the results of using all real datasets; the red line represents the results of using virtual datasets plus real datasets, where the virtual datasets account for 30% of the total datasets. The area near the line represents the random error distribution of the network trained 5 times under the same conditions.

datasets. After training the network with 1,000 groups of virtual datasets, 300 groups of virtual datasets plus 700 groups of real datasets (pre-training and transfer learning), and 1,000 groups of real datasets, respectively, we performed 3D imaging of a static scene consisting of two plaster statues. Figure S7(b) shows the absolute phase error (12-step phase-shifting method and 3-frequency temporal phase unwrapping are used to obtain the ground truth) in three cases. It can be seen that although the network trained only with virtual datasets shows a large error in the measurement of the real scene (MAE = 0.2635 rad), the method combining virtual and real datasets can achieve performance (MAE = 0.1037 rad) close to that of the method using all real datasets (MAE = 0.0872 rad). Moreover, to verify the impact of the number of datasets on the experimental results, we studied the impact of 5 different numbers of datasets (number of datasets = 200, 400, 600, 800, 1,000) on the achieved absolute phase accuracy in Fig. S7(c). The blue line represents the results of using all real datasets; the red line represents the results of using virtual datasets plus real datasets, where the virtual datasets account for 30% of the total datasets. The area near the line represents the random error distribution of the network trained 5 times under the same conditions. As depicted in Fig. S7(c), with a small number of datasets, there is a significant performance gap between the

two methods. However, as the dataset size increases gradually, this gap diminishes. When the number reaches 1,000 groups, the difference is reduced to approximately 0.01 rad. The experimental results prove the effectiveness of the dataset generation technique based on "digital twin" and transfer learning in reducing the dataset acquisition cost in the SSSR-FPP method. In addition, it is worth mentioning that the core of this dataset combination method is the matching degree between the virtual and the real system. In the future, we will further combine physics-informed deep learning methods to further reduce the number of required datasets.

## D. Analysis of spatial resolution and comparison with other methods

In this research, we have constructed a real-world super-resolution dataset by capturing paired LR-HR images of the same scene through adjusting the focal length of a digital camera[13]. It is evident from Fig. S8(a) that there exists an approximately linear relationship between the image plane height $h2$ and the focal length $f$. By increasing the focal length, it becomes possible to naturally capture more intricate details of the scene onto the camera sensor. Moreover, theoretically speaking, selecting a specific value for f enables us to regulate the scale factor, which in turn simplifies the acquisition of high-resolution and low-resolution image pairs across a range of scales.

An imaging system can be modelled as the responding information emitted by the sample, which is diffracted by the optical system and forming an image on the focal plane[14]. Assuming that $I_{\text{in}}(x)$ is the original optical image illuminated onto the detector, the pixel size of the detector array is denoted as $p$, and the pixel pitch is set as $x_d$, the discrete signal captured by the detector can be modelled as:

$$I_d(x) = \left[ I_{\text{in}}(x) \otimes \text{rect}\left(\frac{x}{p}\right)\right] \sum_n \delta(x - nx_d) \tag{S5}$$

$\otimes$ denotes the convolution operation. From a frequency domain perspective, the spectrum of this signal can be interpreted as the spectrum of a low-pass filtered optical image, i.e., a low-pass filtered image formed by the modulation of a sinc function of width $1/p$. According to the Nyquist sampling theorem[15], when the detector pixel size is too large, or the sampling is too sparse, the high-frequency information in the spectrum will be folded along the Nyquist frequency and mixed into the low frequency, leaving it inaccessible for deciphering. The process is graphically represented in Fig. S8(b). The discrete signals captured by the sensor suffer from pixel aliasing (mosaicking) at this point, resulting in a deterioration of the high-frequency information.

In order to ensure sufficient sensitivity, high-speed imaging devices are typically equipped with a larger photoreceptor area of pixels. A larger pixel size could boost light-gathering capabilities, albeit at the expense of reduced spatial resolution caused by decreased sampling density. The spatial sampling frequency of the pixels is suppressed, and the resolution of the imaging system is determined by the Nyquist sampling frequency of the detector pixel size. Therefore, finding an optimal balance between sensitivity and resolution plays a vital role in designing efficient high-speed imaging systems capable of capturing desirable images while maintaining accurate representation of fine details even under challenging conditions or rapid motion scenarios. The proposed imaging system employs a 20 μm pixel size sensor (Phantom CMOS), paired with a 24 mm focal length lens, which theoretically enabled the system to achieve a spatial resolution of 0.833 mrad. To visually depict the phenomenon of information aliasing and spectral degradation resulting from finite pixel dimensions, we present the simulation results obtained using triple down-sampling factors in Fig. S8(b). The whole process demonstrates that the high frequency information is mixed into the low frequency region within the orange rectangle, and the aliasing problem becomes more significant along with the higher sampling factor. For the current pixel size of the sensor (20 μm), pixel aliasing is a pivotal constraint that directly affects the imaging resolution of high-speed systems. We conducted a resolution experiment to quantitatively evaluate the super-resolution performance of our

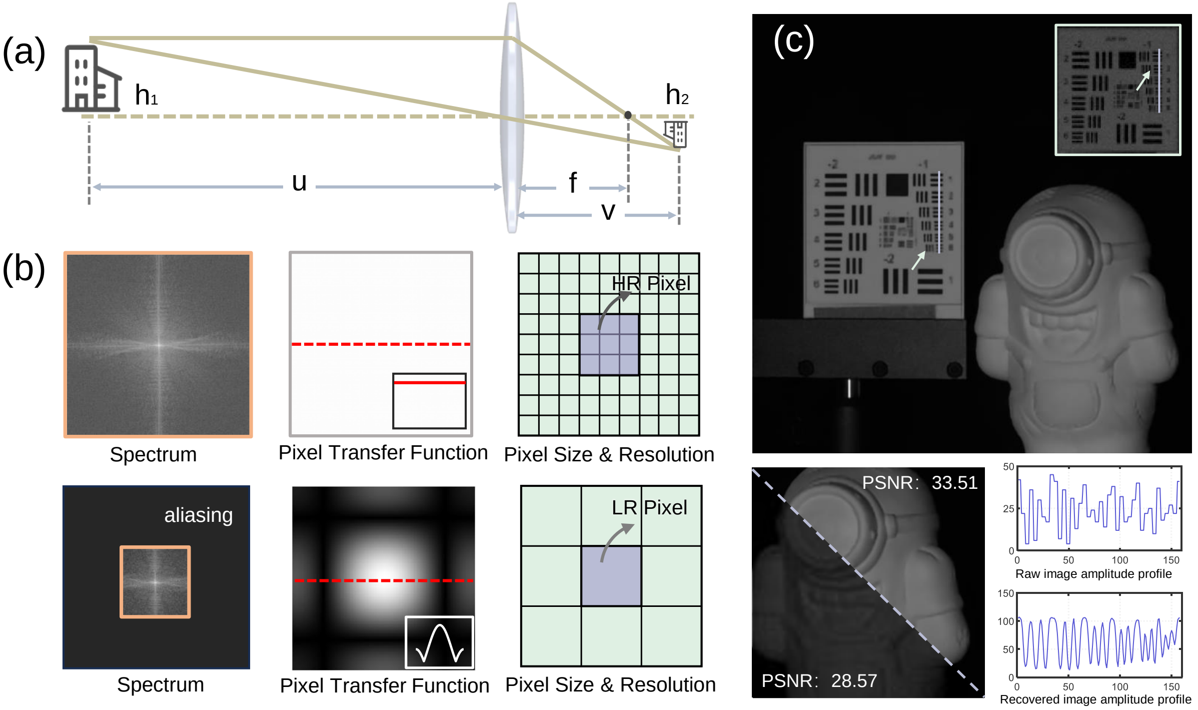


**Figure S8.** Quantitative evaluation of the spatial resolution capability. (a) Illustration of a thin lens, with u, v, and f denoting the object distance, image distance, and focal length correspondingly. The sizes of the object and image are denoted by h1 and h2. (b) Simulation results with triple down-sampling factors are presented along with corresponding Fourier spectrum and pixel aliasing transfer function to illustrate the phenomenon of aliasing. (c) Quantitative evaluation of resolution in experimental results is conducted using standard resolution targets as well as complex objects. A zoomed-in region is assessed for comparison purposes regarding resolvable line pairs and reconstructed signal-to-noise ratio.

proposed method. The USAF resolution chart (75 mm $\times$ 75 mm, Group -2 to Group 7) was positioned at approximate distance of 90 cm from the imaging system. As depicted in Fig. S8(c), the initial resolution of the USAF chart is limited to 0.561-line pairs per millimeter (lp/mm) (Group -1, Element 2). By super resolution resolved, the spatial resolution can achieve up to 0.891 lp/mm (Group -1, Element 6), giving a factor of 1.58 $\times$ improvement in resolution. Combined with the prior knowledge of the network model, as shown in Fig. S8 (c), the resolution of the targeting results is improved and the details on the plaster model become sharp, in conjunction with the improvement of the signal-to-noise ratio of the resolved image from 28.57dB to 33.51dB.

To demonstrate the rationality and effectiveness of our constructed deep learning network and quantitatively evaluate the accuracy of reconstruction phase information, we employed the same training datasets and compared the performance of different network structures (MVSNet[16], DCNN[17], Multi-path CNN[18], etc.) with our method in a comparative experiment. Figure S9(a) shows the absolute phase errors under the different methods (the ground truth is obtained using the 12-step phase shifting method), respectively, and as expected from our previous analyses, the phase information of the smoothed region can be retrieved accurately. In contrast, phase measurements in complex regions exhibited singularities.

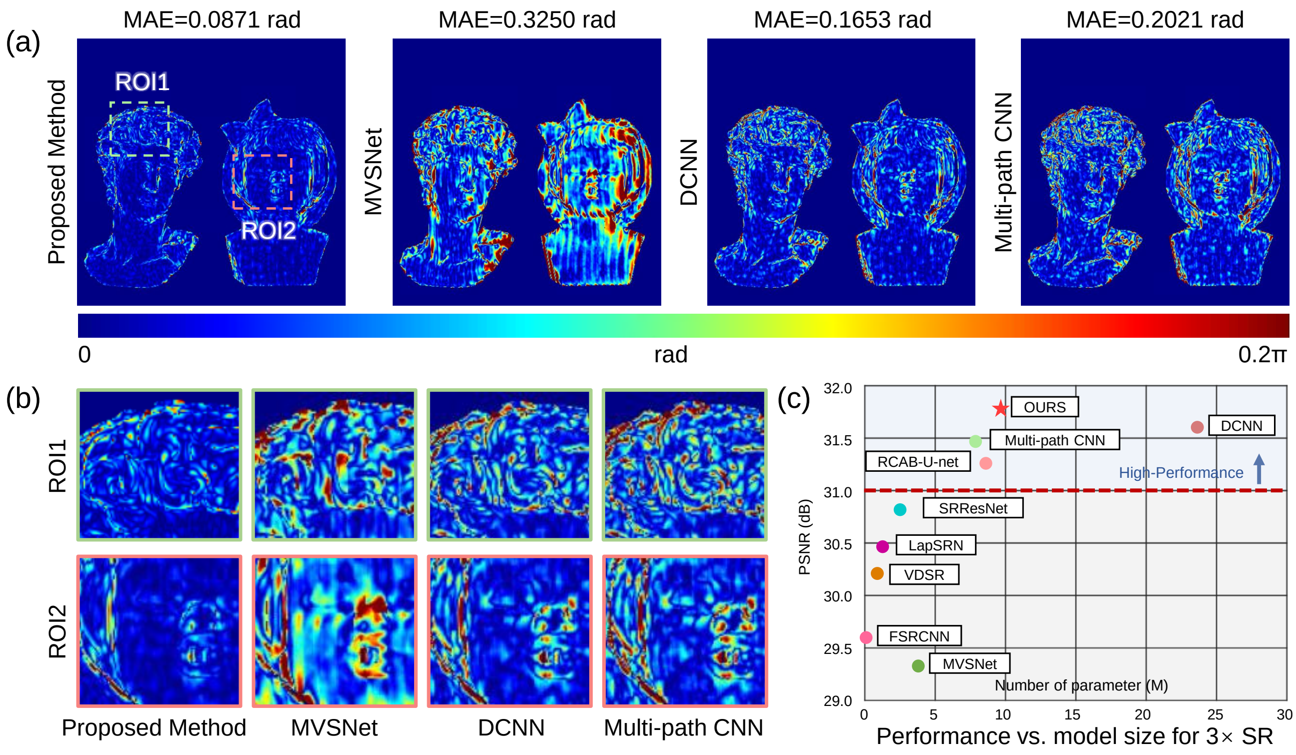


**Figure S9.** Comparison of reconstruction results and model parameters with different network structures. (a) The absolute phase error maps of the MVSNet, DCNN, Multi-path CNN, and our method, respectively. (b) Selected ROIs of the phase error for the four methods. (c) Comparisons of the performance and the number of parameters among different network structures.

For a comprehensive analysis, two specific regions of interest (ROI) were selected within the building model. It is evident that our approach outperforms MVSNet, DCNN, and Multi-path CNN significantly when dealing with intricate areas characterized by depth variations and edges. In terms of quantitative evaluation, the mean absolute error (MAE) is substantially reduced to 0.0871 rad through the utilization of our method, showcasing an impressive 47% enhancement in accuracy compared to the DCNN network.

In the proposed method, the framework of CNN1 combines a dual regression architecture and a composite loss based on both physical and data. The dual regression architecture establishes a mutual mapping between LR images and HR images, which corresponds to the fact that the network can take into account both forward and inverse models for image resolution enhancement. The composite loss enables the network to be driven by both data and physical models and is able to adaptively learn the "physically meaningful" prior knowledge implicit in real experiments. This specially designed architecture improves the network robustness and the generalization capability under real-world settings. Meanwhile, the CNN1 architecture incorporates a physical model of the phase-shifting method, which can bypass the difficulty of predicting $2\pi$ jumps in the wrapped phase function, thereby improving the accuracy of the reconstructed phase effectively. In addition, we design CNN2 to organically unify geometric constraints,

and phase unwrapping into a comprehensive framework. It can decipher the absolute phase by combining the information from the reference plane. Although the absolute phase output is relatively ”coarse” due to the environmental light, large surface reflectivity, and discontinuities, it is sufficient to solve the precise fringe order of the wrapped phase, which preserves both the high precision of the wrapped phase obtained by CNN1 and the high accuracy of the fringe order obtained by CNN2 and thus ensures a high-resolution and high-precision absolute phase.

We further present a graphical comparison in terms of performance and model size for 3× super-resolution in Fig. S9(c). In the scatter diagram, the vertical coordinate represents the PSNR of the predicted graph, and the horizontal coordinate denotes the network model parameters. MVSNet is an end-to-end deep learning architecture built on the principle of multi-view stereo (MVS). MVSNet can extract deep image features and apply 3D convolutions to generate the final output. This architecture performs well when receiving images from multiple different viewpoints, but in ultra-high-speed scenarios, cameras capable of capturing high-speed motion scenes are typically expensive. In this work, our optical setup is only able to provide a pair of fringe images (2 viewpoints), which leads to the degradation of the MVSNet reconstruction accuracy. The DCNN utilizes traditional up-sampling algorithms to obtain high-resolution images and improves them by learning end-to-end mapping from interpolated coarse images to high-resolution images of the same dimensions, as depicted in Fig. S9(c). However, a significant drawback of DCNN lies in the time and space overhead associated with maintaining the entire resolution throughout the network, thereby limiting its applicability to relatively shallow network architectures. Multi-path CNN is a deep neural network composed of multiple paths (here the number of paths is 4), each path consists of multiple convolutional layers and a group of residual blocks, performing encoding, feature extraction, and decoding of input data. Multiple paths can effectively mitigate the accuracy degradation problem when the network becomes deeper. However, the lack of consideration of physical models in the Multi-path CNN architecture limits its reconstruction accuracy in super-resolved fringe analysis. Compared with several state-of-the-art networks, we observe that the SSSR-FPP method introduces a regression structure to flexibly cope with the dilemma of a large solution space for mapping functions from low-resolution images to high-resolution images under non-deep networks. The proposed method demonstrates superior performance in terms of accuracy and efficiency and achieves remarkable performance with a relatively sparse parameter set. Both theoretical analysis and experimental results show the effectiveness of the proposed SSSR-FPP scheme and achieve the state-of-the-art speed-accuracy trade-off.

To highlight the unique value of the SSSR-FPP method in ultra-high-speed imaging tasks, we analyzed and compared the performance of the proposed method with three mainstream 3D reconstruction techniques in five aspects: imaging speed, number of point clouds, accuracy, independence from calibration, and cost-effectiveness. In addition, we drew a radar chart as shown in Fig. S10.

- The first technique is structure from motion (SfM)[19]. SfM reconstructs 3D scenes by analyzing the motion and features across multiple 2D image frames, enabling the creation of 3D models from image sets. This technique usually requires a moving camera to continuously capture 2D images of the reconstructed scene (usually static), and is mainly used in mapping, visual navigation, and other fields. SfM has low imaging speed and accuracy. Because it can calculate the internal and

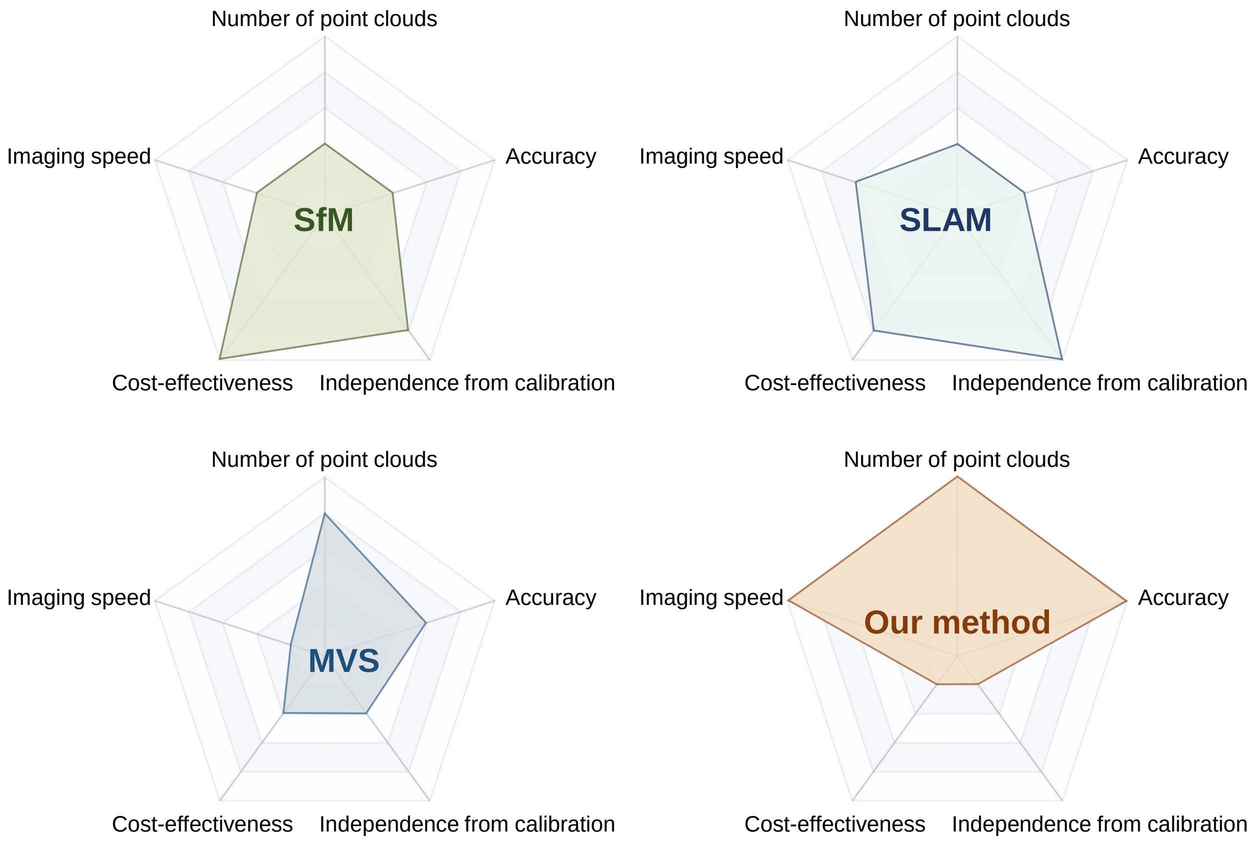


**Figure S10.** The comparative performance of the SSSR-FPP method and three mainstream 3D reconstruction techniques (SfM, SLAM, and MVS) in terms of imaging speed, number of point clouds, accuracy, independence from calibration, and cost-effectiveness.

external parameters of the camera through feature matching, it does not rely on pre-calibration, but the number of reconstructed point clouds is relatively sparse, and the advantage is high cost-effectiveness.

- The second technique is simultaneous localization and mapping (SLAM)[20]. SLAM is a technique that enables a device to map its environment while simultaneously determining its own location in real time. It is commonly used in robotics, autonomous vehicles, and augmented reality systems. SLAM is similar to SfM, but the measured scene can move (slow speed) and it focuses more on real-time reconstruction. The imaging speed of SLAM is higher than SfM, but the accuracy and number of point clouds are relatively low. It is cost-effective and does not rely on calibration.
- The third technique is multi-view stereo (MVS)[21,22]. MVS involves reconstructing 3D scenes by combining information from multiple 2D images taken from different viewpoints, enabling the recovery of more detailed depth information. MVS requires multiple 2D images from different perspectives (usually $> 3$) to reconstruct scenes (static), and this process requires pre-calibration of the camera. Its imaging speed is very low, but the accuracy and number of point clouds are higher

than SfM and SLAM. As the reconstruction accuracy and number of point clouds increase, the cost-effectiveness of MVS decreases.

- The fourth technique is the SSSR-FPP method proposed in this work. Due to the artificial projection to help correlation matching (which is not available in the above three techniques) and the ability of super-resolution imaging, SSSR-FPP can work in the ultra-high-speed imaging mode and can perform high-precision phase recovery and dense 3D reconstruction of transient scenes at a speed of 100,000 fps. It requires the parameters of the camera and projector to be calibrated in advance. Compared with the above three techniques, this method has obvious advantages in imaging speed, accuracy, and number of reconstructed point clouds.

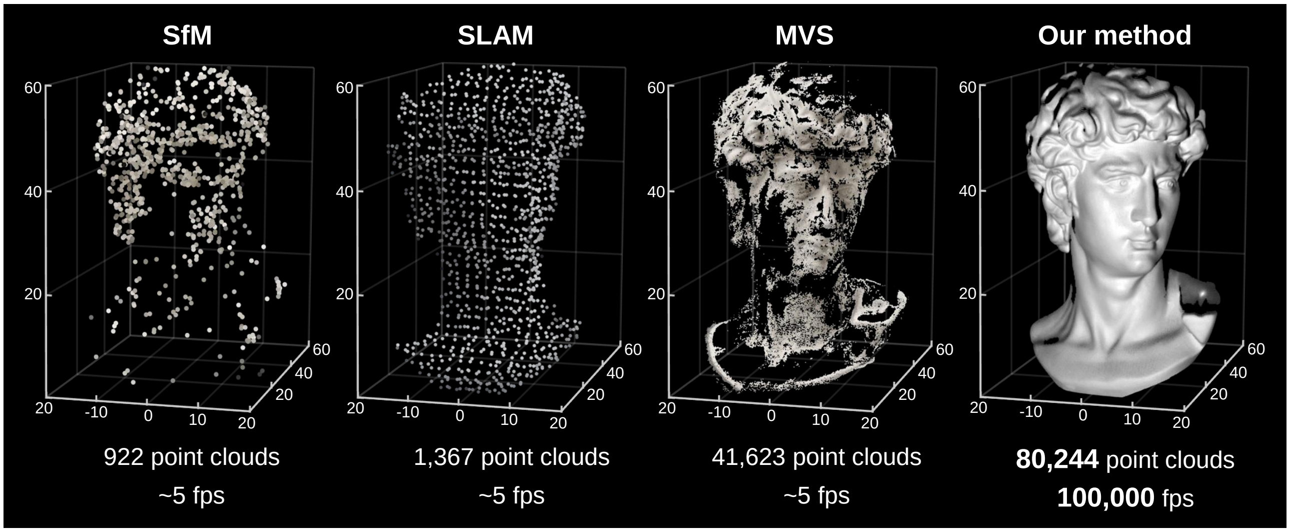


**Figure S11.** A comparative experiment on 3D reconstruction of a static plaster statue using SfM, SLAM, MVS, and our method, providing a qualitative comparison of reconstructed accuracy and a quantitative comparison of point cloud number and imaging speed.

Furthermore, we conducted a comparative experiment to highlight the advantages of our method over other existing techniques. Specifically, we tested 3D reconstruction performance on a static plaster statue of David using SfM, SLAM, MVS, and our proposed method. It is important to note that since SfM, SLAM, and MVS require multiple 2D images for single-shot 3D point cloud recovery, we used 20 images for each of these techniques in the experiments. This multi-frame requirement significantly limits the imaging speed of these methods; when using conventional cameras, the maximum achievable frame rate is typically around 5 fps (as shown in Fig. S11). As further depicted in Fig. S11, the SfM method was only able to extract a small number of feature points and lacked accurate camera calibration, resulting in low reconstruction accuracy and a sparse point cloud (922 points). Similarly, the SLAM method, which prioritizes real-time reconstruction, also produced a limited point cloud (1,367 points) due to computational constraints, and the reconstruction accuracy remained low. MVS, benefiting from pre-calibrated parameters, achieved higher accuracy and a significantly larger point cloud (41,623 points)

compared to SfM and SLAM. In contrast, our method demonstrated significantly superior performance, achieving high-precision 3D reconstruction with 80,244 points and an unprecedented imaging speed of 100,000 fps. These results underscore the major advantages of our approach over traditional techniques. The experimental outcomes highlight the tremendous potential of our method in ultra-high-speed 3D imaging, making it a powerful tool for investigating transient phenomena. Our method could offer valuable insights into the physical mechanisms behind such events, with broad applications in fields such as aerospace, biomedicine, and national defense. In addition, it is worth mentioning that although the reconstruction indicators of SfM, SLAM, and MVS can be improved by deep learning, from the existing technical solutions, our method has obvious performance advantages in imaging speed, number of point clouds, and accuracy when measuring single-sided objects. Therefore, the SSSR-FPP will owe more advantages when further applied to 360-degree 3D shape measurement.

## E. Analysis and comparison of different fringe pattern schemes

In addition, we analyze the phase information decoupling ability of different fringe pattern schemes in the high-speed imaging system. Figure S12(a1-a2) and (b1-b2) show the fringe images ($480 \times 480$) captured at an exposure time of 45 µs under different designed fringe patterns and the corresponding spectral images, respectively. Figure S12(c1) and (d1) present the fringe images ($160 \times 160$) captured at an exposure time of 9.5 µs in different projection modes, respectively. With the increasing number of composite frequencies in the fringe pattern, the modulation intensity of the fringe image is gradually weakened. Therefore, the fringe modulation information present in a single-frequency fringe image is substantially greater than that observed in composite fringe image. With the increase in a camera's maximum frame rate, the exposure time is too limited to acquire a sufficiently illuminated image (in this paper, the exposure time is set to 9.5 µs), resulting in images with poor SNR and evident read noise superimposed. It is evident that the large pixel size of the high-speed camera, coupled with the employment of a low-focal-length lens, leads to significant spectral aliasing, resulting in the loaded composite image is difficult to distinguish. In this case, the composite fringe information will be invalid due to the presence of spatial aliasing and lower fringe modulation information. Designed networks faced a challenging task of separating the correct frequency components and solving the wrapped phase from an aliased spectrum, even in the presence of readout noise. Conversely, the single-frequency fringe pattern can preserve the embedded information to the maximum extent.

Figure S12(e) displays the reconstruction results obtained with a composite fringe network. However, due to the poor quality of the original fringe image, the network troubled to accurately estimate the absolute phase orders, resulting in incorrect 3D reconstruction results. Additionally, we have presented the 3D rendered geometry obtained through the widely recognized phase-shifting profilometry technique, as shown in Fig. S12(f). Upscaling of the original fringe images can yield higher-quality 3D reconstructions that more effectively retain surface details. Nonetheless, inaccurate interpolation may result in non-sinusoidal waveforms, potentially causing periodic phase errors. Despite the presence of background noise in the original fringe images, the proposed method effectively restores the absolute phase information, which is converged with the ground truth (result obtained using a 3-frequency, 12-step phase-shifted unwrapping method). Our approach breaks through the imaging system's Nyquist sampling constraints, providing a precise depiction of the facial 3D geometry, presented in Fig. S12(g). We performed a quantitative analysis utilizing SSIM/PSNR metrics, demonstrating that our method significantly enhances the SSIM by 0.12 and boosts the PSNR by 9.01 dB compared to the output reconstruction result of multi-frequency composite fringe network.

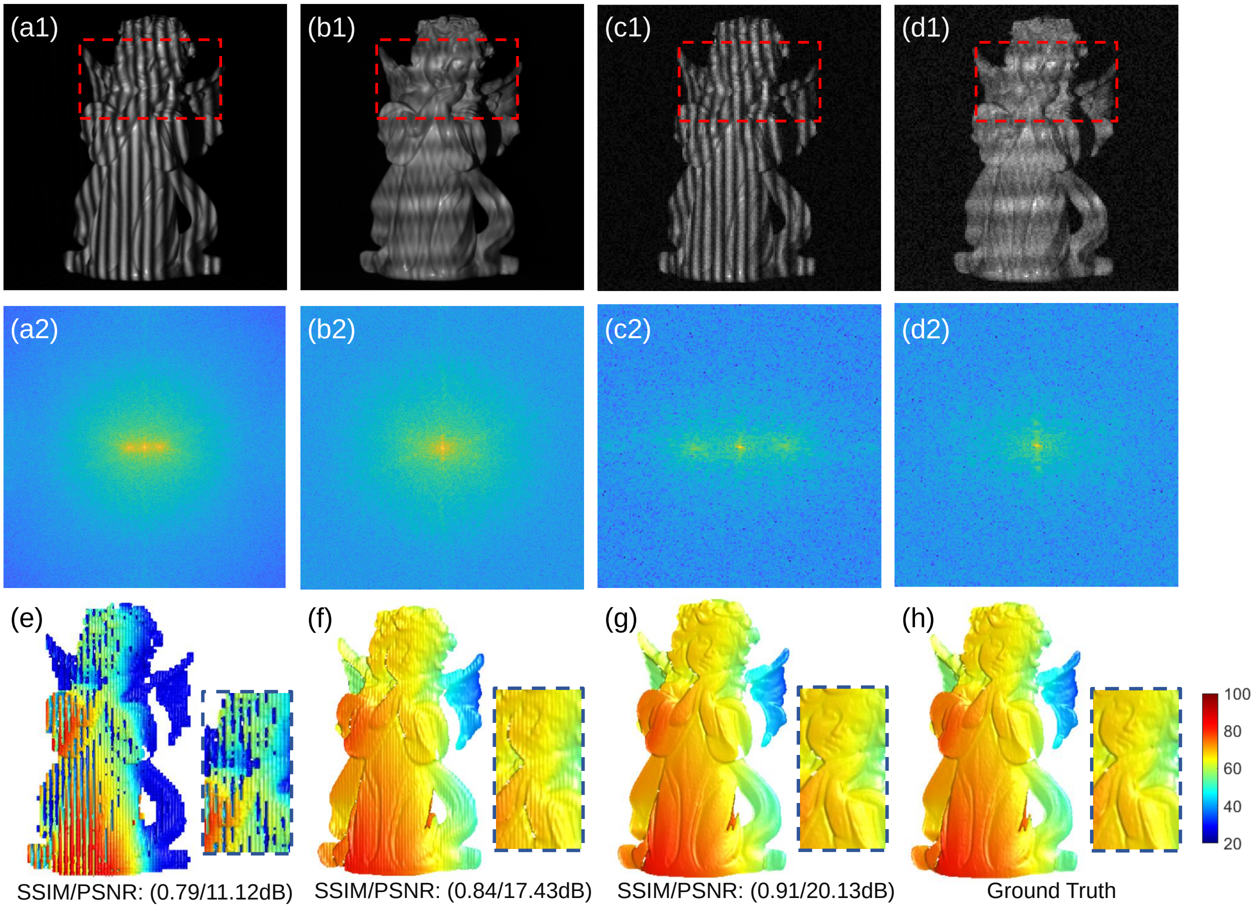


**Figure S12.** Analysis of different fringe pattern schemes and corresponding resolved results. (a1, b1) High-resolution single frequency and multi frequency fringe image (72 mm, 45 μs, raw image resolution $480 \times 480$). (c1, d1) Low-resolution single frequency and multi frequency fringe image (24 mm, 9.5 μs, raw image resolution $160 \times 160$). (a2-d2) The corresponding spectrum of the different designed fringe images. (e) Output reconstruction result of multi-frequency composite fringe network (The fringe image fed into the network is shown in Figure d1). (f) Reconstruction results generated by a 3-frequency and 3-step phase-shifting method using the nearest interpolation image. (g) Output reconstruction results of the proposed method (The fringe image fed into the network is shown in Figure c1). (h) 3-frequency and 12-step phase-shifting unwrapping method reconstruction result, denoted as the ground truth (raw image resolution $480 \times 480$).

## F. Measurement results of the diverse static plaster models

To test the phase demodulation performance of the trained network, we apply SSSR-FPP method to demodulate the fringe images obtained by measuring different types of samples. we compared the 3D reconstruction results obtained by our method with those generated from raw low-resolution fringe patterns using various up-sampling strategies (Note that in order to obtain absolute phase, these methods still require 3-frequency and 3-step phase-shifting measurements, i.e., 9 fringe patterns in total, the only difference is that the exposure time is 45 μs). It can be observed that as the exposure time increases, the signal-to-noise ratio of the reconstruction rises significantly, and the contrast is upgraded. However, it is often necessary to consider the trade-off among imaging speed, exposure time, and imaging resolution in practical imaging. Unfortunately, it is a paradox to simultaneously obtain both a high signal-to-noise ratio and ultra-fast imaging frame rate image. The comparison of phase unwrapping results between the phase shifting method and the deep-learning-based method is illustrated in Fig. S13, indicating that the deep learning method can directly execute complex nonlinear phase unwrapping task with improved anti-noise and anti-aliasing capabilities. As demonstrated in Fig. S13(b), even if the 3-frequency and 3-step phase shifting method is adopted, it is still challenging or almost impossible to reconstruct a 3D imaging result with textured information and decent shape at the frame rate of high-speed imaging (short exposure and low resolution).

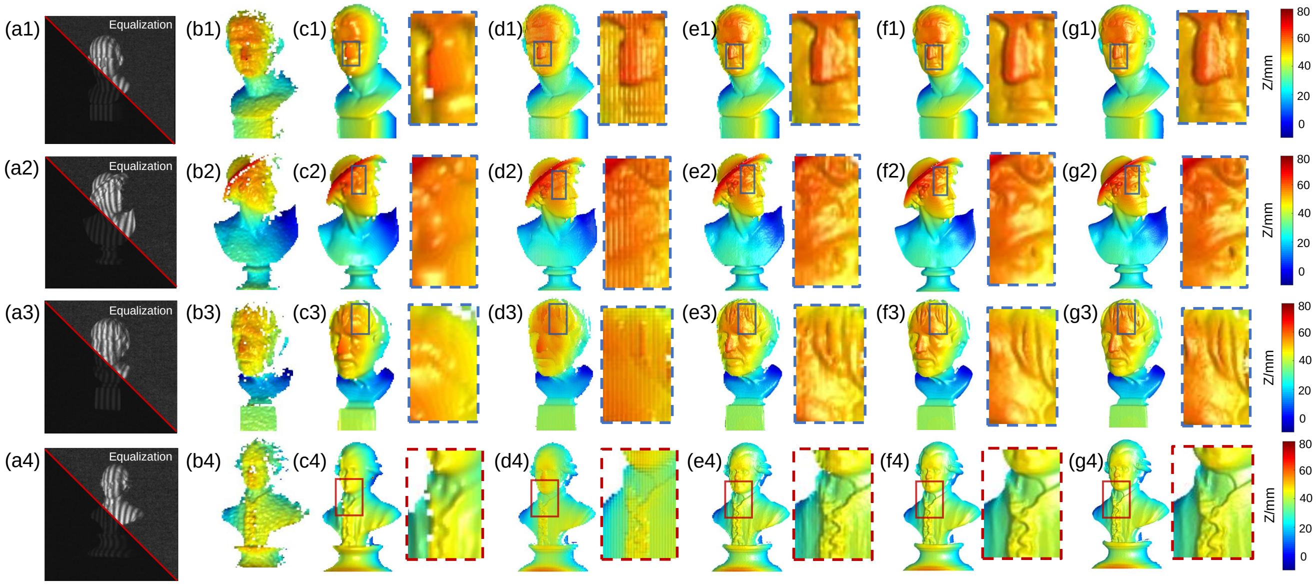


**Figure S13.** Comparison 3D measurement results of diverse static plaster models generated by different methods. (a) Low-resolution fringe image. (b) The low resolution 3D reconstruction results (9.5 μs). (c) The low resolution 3D reconstruction results (45 μs). (d) The nearest interpolation 3D reconstruction results (45 μs). (e) The bicubic interpolation 3D reconstruction results (45 μs). (f) 3D reconstruction results generated by our network (9.5 μs). (g) The ground truth (High resolution 3-frequency 12-step phase-shifting unwrapping method result, denoted as the ground truth, 45 μs).

Even though a high exposure (45 μs) low-resolution image is fed into the traditional phase-shifting method, the high-resolution texture information is yet to be effectively restored by using the traditional interpolation method, as depicted in Fig. S13(c). In contrast, our proposed algorithm can still reconstruct the three-dimensional morphology of the approximate ground truth image when the resolution and signal-to-noise ratio is unbalanced, as depicted in Fig. S13(f). In this scenario, the phase retrieval problem is effectively addressed, particularly as it provides higher fidelity texture information compared to classical methods, especially noticeable in the details of hair, eyes, and collar on a static plaster model. Different from the multi-frame phase-shifting method, such high-quality phase demodulation is obtained from only one pair of fringe images as input. In addition, unlike the Fourier transform method, where the performance heavily relies on the fine-tuning of several parameters, the deep learning method is fully automatic — once a network is completed training, it removes the requirement for any manual parameter adjustments to optimize its performance.

## G. Dynamic 3D measurement results in multiple scenarios

### Analysis of rotating fan blades

The extraction of phase information with the highest accuracy, fastest speed, and full automation continues to be a primary focus in the field of optical metrology. In this context, we demonstrate single-shot imaging capability by introducing a 3-bladed commercial fan experiment. Although the fan is operating at its maximum speed, the 10 μs exposure time of SSSR-FPP system is short enough to freeze the high-speed motion and produce high precision 3D measurement results of the whirling fan blades, as shown in Fig. S14.

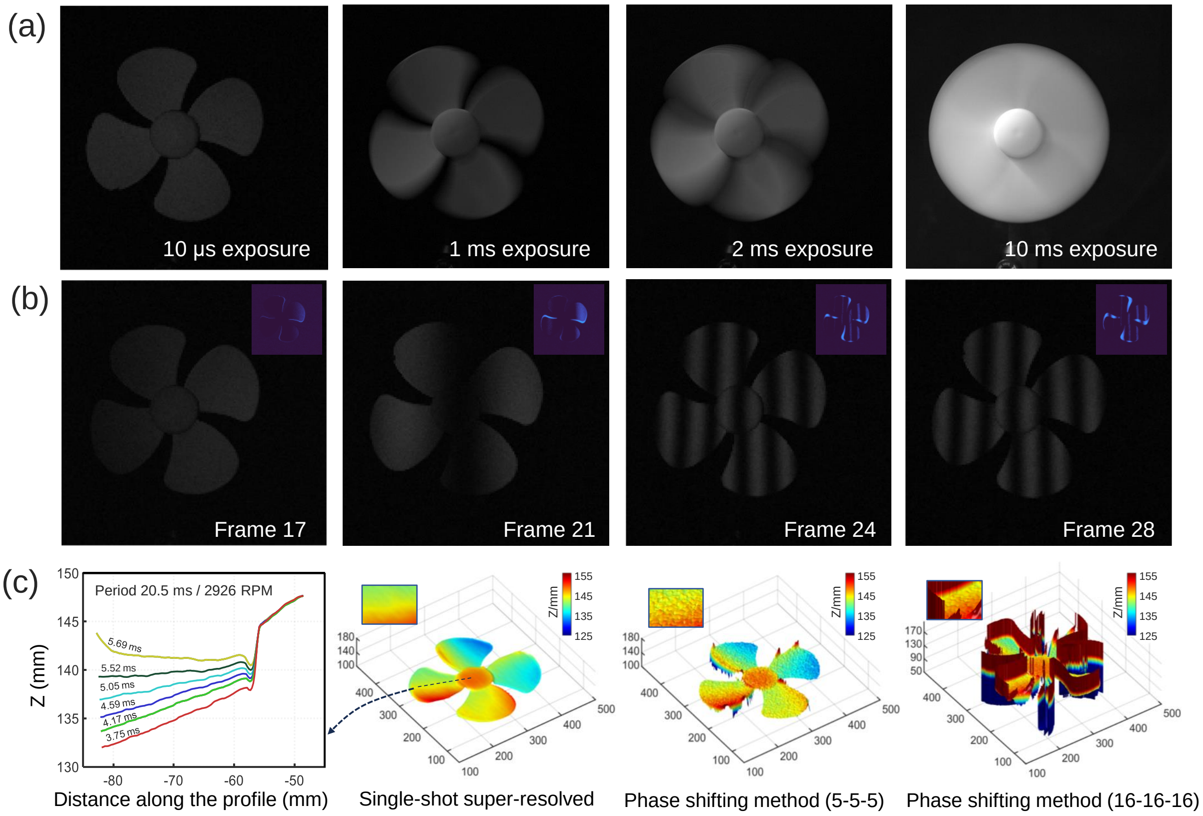


**Figure S14.** Measurement of rotating blades. (a) Images captured by the camera at different exposure time (10 μs, 1 ms, 2 ms, and 10 ms). (b) The images collected at various frames were presented individually, revealing a discernible trend of increasing displacement error between the collected images and the initial frame image as time progresses. (c) The 3D imaging results of the traditional method and the proposed method at a typical moment. The upper left corner of each subfigure displays a magnification of the selected region of interest in the original image.

To visually demonstrate the rotational speed of the fan blades in a more intuitive manner, we modified the camera exposure time from 46 μs to 1 ms, 2 ms, and 10 ms. With the increase in exposure time, the edges of the fan blades are gradually blurred. The previously sharp and well-defined contours gradually diminished in clarity. As the exposure was gradually increased to 10 milliseconds, we failed to distinguish

the fan blades suffering from the effects of motion blur. To further analyze the SSSR-FPP system's ability to circumvent the disturbance of motion artifacts in dynamic scenes, we compared the proposed method with the traditional phase shifting with stereo phase unwrapping method. As can be seen in Fig. S14(b), the inherent multi-fringe property of the phase-shifting method, which naturally requires trading off a certain amount of temporal resolution, feeding back as causing motion artifacts/blurring between images at regular intervals. The images collected at various frames were presented separately, revealing a discernible trend of increasing displacement error between the collected images and the initial frame image as time progresses. Therefore, due to the inherent constraints of multi-frame projection, existing physically based multi-frame projection methods fail to produce high-quality, artifact-free 3D data if moving objects are present between each frame. As highlighted in the zoom area, the reconstruction results obtained through the traditional phase-shifting method present evident artifacts and unwrapping errors near sharp edges. In contrast, SSSR-FPP yields a significantly smoother reconstruction devoid of notable artifacts. Despite the challenging rotation speed, the SSSR-FPP method successfully reconstructed the 3D shape of the entire fan, including the center hub and three blades, as depicted in Fig. S14(c). It can be concluded that the rotation period of the fan is about 20.5 ms, which corresponds to a speed of 2,926 revolutions per minute (rpm). The plot curve also demonstrates a decent repeatability of the SSSR-FPP measurement. More precisely, these results indicate that SSSR-FPP notably improves the performance of the accuracy and robustness of state-of-the-art methods for measuring textured surfaces.

From the comparison results, it can be witnessed that the proposed learning-based reconstruction method is immune to motion blur and elegantly reconstructs the object's high-precision 3D profile owing to the native advantages of single-frame projection. In contrast, conventional methods fail to eliminate the motion disturbances caused by phase shifting, thus generating jump errors and noticeable ripples on the reconstructed surfaces. With low signal-to-noise ratios induced by readout noise from extremely short exposures under high-speed photography, the SSS-FPP method can break the physical imaging confinement to decipher high-quality reconstruction results, which are vulnerable to other methods.

## Analysis in the non-repetitive transient event

We demonstrate the application of SSSR-FPP to a non-repetitive transient event: The bullet was fired from a toy gun, hitting a flat plate and subsequently rebounding. Figure S15(a) illustrates color-coded 3D reconstruction results recorded at various time points. The observation initiates at T = 0 ms, and around T = 4.81 ms, the bullet can be found near the muzzle. After a free flight of approximately 15.17 ms, the bullet collided with the plate and rapidly rebounded. Figure S15(b) presents a 3D reconstruction of the plate's dynamic response to the bullet impact, showcasing its tilting motion process. Additionally, figure S15(c) depicts the trajectory of the bullet at distinct moments. The 3D shape of the bullet was frozen at 7.76 ms, and by analyzing the horizontal (x-z) and vertical (y-z) cross-sections of the bullet, we can conclude that the length of the bullet is approximately 29.4 mm and the diameter is about 11.2 mm. In addition, the inset plots the bullet velocity as a function of time. The 3D measurement data enables a precise quantitative analysis of the bullet's ballistic trajectory and velocity characteristics. The instantaneous velocity of a bullet is determined by extrapolating its position in relation to time, yielding an initial velocity of approximately 9.45 meters per second as it leaving the barrel of the gun. The advantage of SSSR-FPP is that phase information is encoded in a single fringe pattern, which eliminates artifacts in frame-by-frame motion. A more detailed illustration of transient events is provided in Supplementary Video 4. Provided experimental results demonstrate the potential application of the SSSR-FPP for tracking the 3D trajectories of fast-moving objects over broad observational ranges.

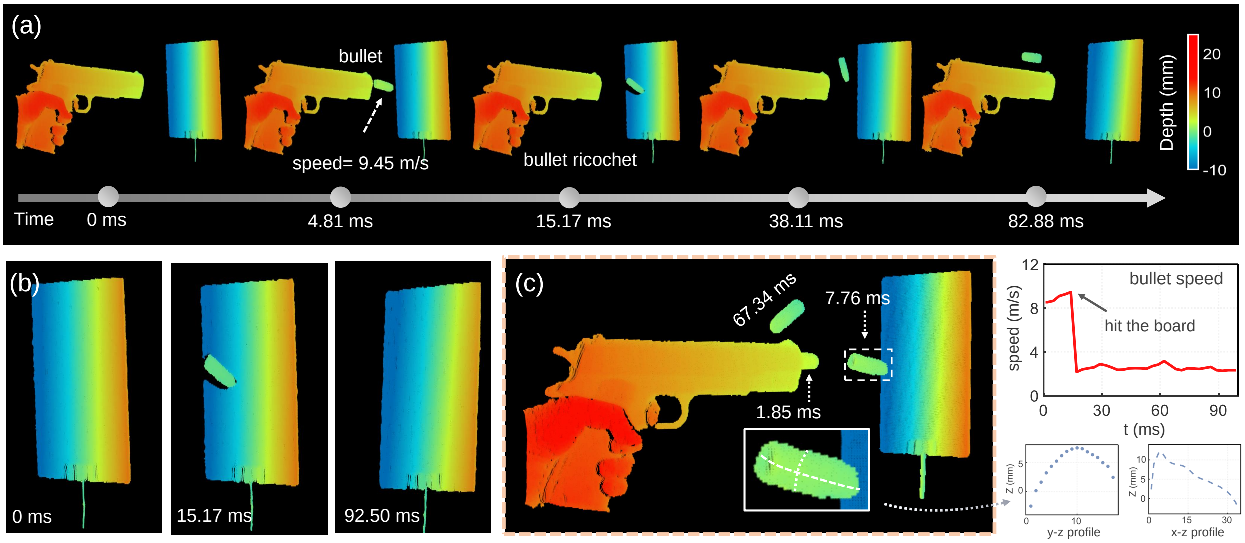


**Figure S15.** The 3D measurement results of a bullet fired from a toy gun and subsequently hitting the flat plate. (a) Representative color-coded 3D reconstructions at various time points. (b) As the bullet impacted the flat plate, kinetic energy was transferred, causing the plate to tilt backwards. At each moment the angle variation of the flat plate was recorded. (c) 3D reconstruction of the muzzle area, along with the bullet's trajectory at three distinct time points during flight (1.85 ms, 7.76 ms, and 67.34 ms). The inset displays the horizontal (x-z) and vertical (y-z) cross-sections through the center of the bullet at 7.76 ms. In addition, the inset plots the bullet velocity as a function of time.

## Analysis in the physical transformation process of a steam engine

In order to further evaluate the effectiveness of the SSSR-FPP system in practical applications, we analyze the physical motion process of the steam engine by 3D reconstruction (See full version in Supplementary Video 5). We selected a steam engine with actual dimensions of 16.5 cm × 9.5 cm × 13 cm. By continuously heating the tube at a high temperature, high-pressure steam is generated and transported to the cylinder through the pipe. Inside the cylinder, the steam pushes the piston in reciprocating linear motion, which is connected to the crankshaft through a connecting rod. The main characteristic of the crankshaft is to convert the linear reciprocating motion of the piston into a rotating motion of the crankshaft.

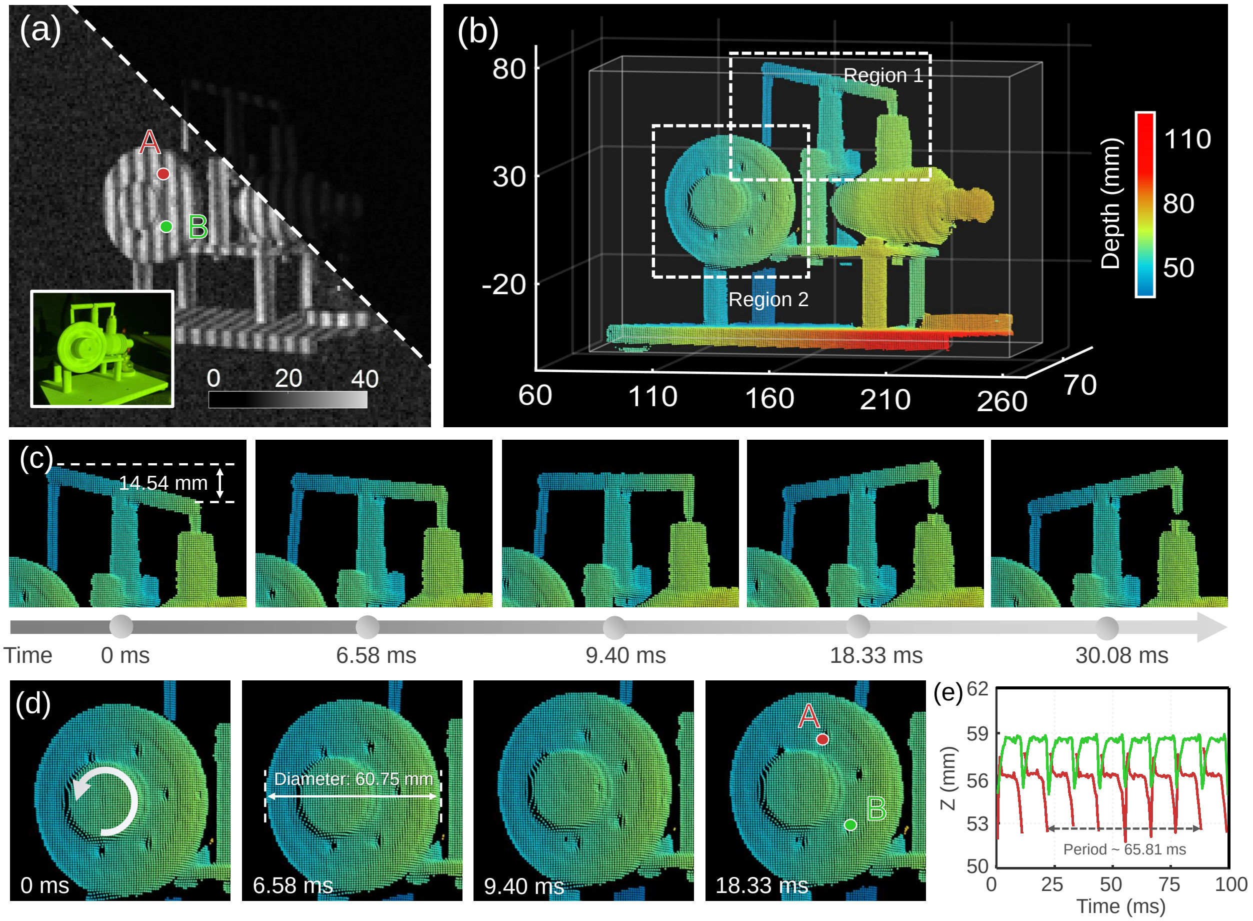


**Figure S16.** The 3D measurement results of the reciprocating motion of a steam engine's piston driven by steam. (a) The raw image obtained by the camera. (b) At the beginning of the observation period (T = 0 ms), the steam engine 3D shape was remodeled. (c) The color-coded rendering of 3D reconstruction results, frozen at different moments, is shown for the selected region 1. (d) The color-coded rendering of 3D reconstruction results for the selected region 2. (e) Displacement variation in the z-direction at two picked point locations [A and B, as presented in (a)] over a time function of 100 ms.

The raw captured image is depicted in Fig. S16(a). Even with the presence of background noise and pixelation in the original image, the SSSR-FPP technique effectively delineates the complete 3D

geometry of the steam engine. Concurrently, we illustrate the three-dimensional motion of two distinct regions at various moments, capturing both reciprocating linear and rotational movements. By tracking and analyzing the motion process of the connecting rod, we can obtain the stroke of the piston as 14.54 mm. The diameter of flywheel was precisely measured to be 60.75 mm through optimal spherical fitting. To verify the data reproducibility, we have randomly selected two points on the flywheel, labeled as A and B in Fig. S16(a), to demonstrate the cycle of motion. Referring to Fig. S16(e), the vertical displacement in the z-direction at the specified locations over 100 ms duration is charted. We can calculate that the rotational period of the fan is about 65.81 ms, indicating that the SSSR-FPP measurements are consistently repeatable. The dynamic structure and motion process inside the steam engine can be demonstrated in detail by 3D visualization technology, enabling the observer to intuitively comprehension of its operational dynamics and the mechanisms of energy conversion.

## Analysis of free-falling water balloons and falling building blocks

To validate the reconstruction capability of the SSSR-FPP for complex deforming objects, we recorded 3D measurements results of a free-falling water balloon in Fig. S17(a). After free-falling about 105 ms, the balloon reaches the ground while being significantly squeezed and deformed, losing its original smooth shape (T = 122.43 ms). Due to the ground reaction force, the balloon starts rising and gradually returns to its previous appearance (T = 165.32 ms). The surface of the balloon is elegantly reconstructed throughout the imaging capture process, demonstrating the reliability of the proposed method in high-precision absolute 3-D shape measurement. Similarly, figure S17(b) illustrates the color-coded 3D reconstructions of freely falling building blocks, with a duration of 0.35 seconds, that are stacked into six layers. It is challenging for traditional methods since the scene encompasses random discontinuous edges, such as building blocks and steep edges, which can be revolutionized with our SSSR-FPP method. It is revealed that SSSR-FPP method can precisely reconstruct multiple objects with steep edges and random motion. The corresponding 3D rendering results are provided in Supplementary Videos 6 and 7. The provided experimental results verifies that our method can offer high-quality 3D measurements with fewer exposure time and faster recording speeds. The developed and trained deep neural network demonstrates that it can accomplish phase demodulation tasks accurately and efficiently, using only a pairs of single fringe images. Thus, SSSR-FPP promises to facilitate new measurement capabilities to study various super-fast dynamics scenes and evolve our knowledge in different disciplines.

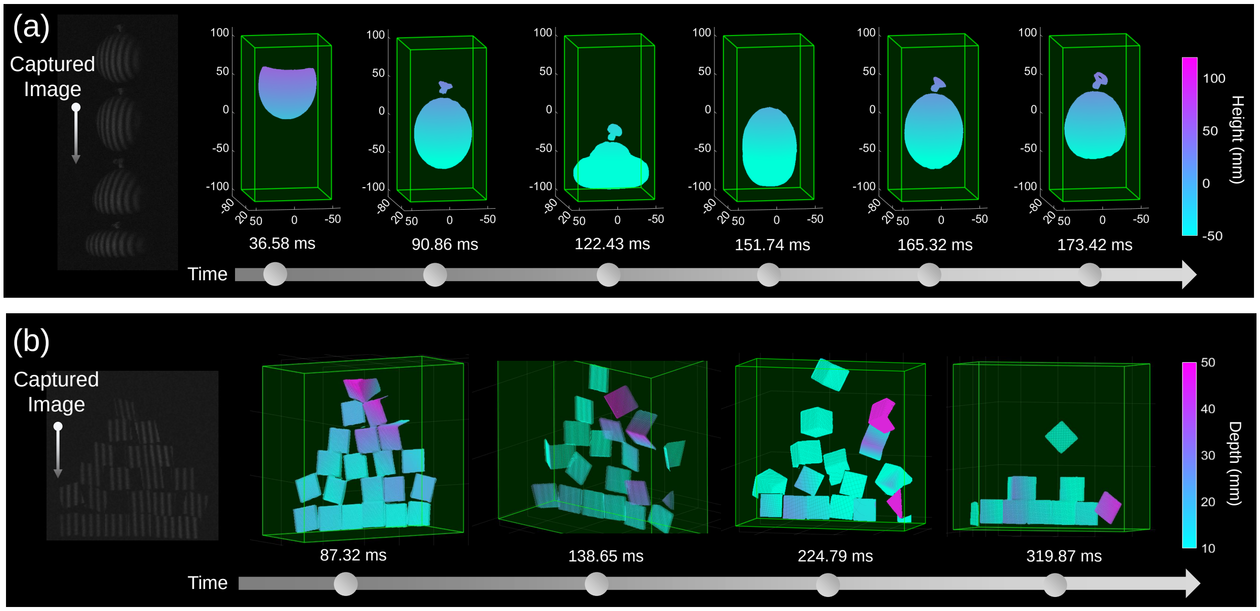


**Figure S17.** The color-coded 3D reconstruction of a falling balloon and the falling building blocks at different times.

### Analysis of 3D reconstruction results for objects with different materials and colors

In the former experiment, we focused on the dynamic measurement capabilities of the proposed method. The cameras are working at a reduced image resolution (160 × 160) so that it is capable of capturing consecutive images at a frame rate of 100,000 fps with the exposure time of 9.5 µs. Constrained by the drastically shortened exposure time, the phenomenon of over-exposure is almost unattainable for normal scenarios, and instead manifests as a low signal-to-noise ratio. Due to the polychromatic nature of the target object, different materials and coloured surfaces would produce different responses to the projected light which in turn causes a variation in the contrast of the modulation intensity within fringe images.

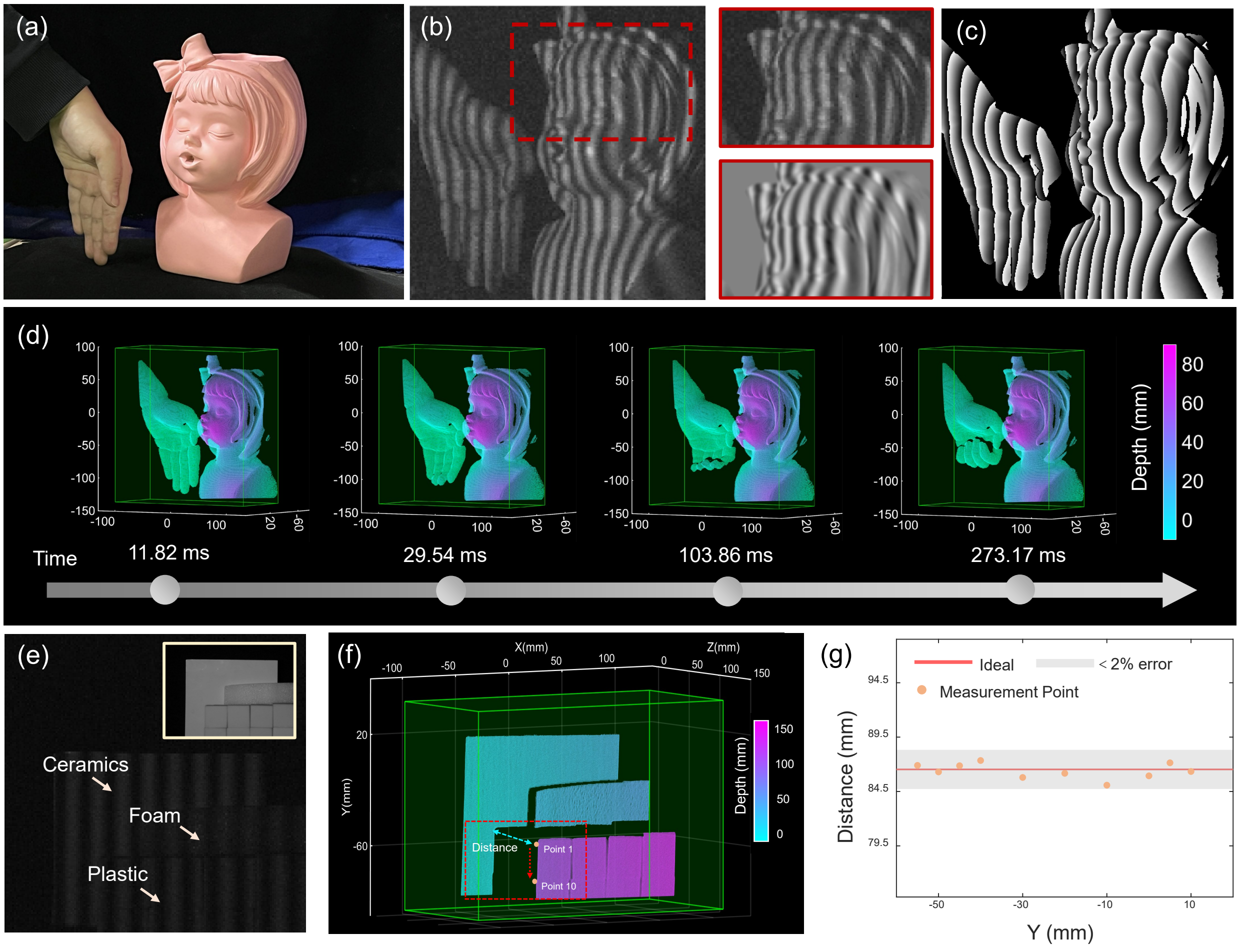


**Figure S18.** Multi-chromatic object 3D reconstruction results. (a) Unprojected colored object captured by a color camera. (b) Low-resolution fringe image, which serves as the raw image input for the network, along with its corresponding predicted "Numerators". (c) The predicted wrapped phase. (d) Color-coded 3D reconstructions at different time points in dynamic scenes. (e) The raw image captured at varying depths, showcasing the established scene adorned with diverse materials including plastic, foam, and ceramics. (f) The reconstructed 3D point cloud data under different materials. (g) Scatter plot of depth error between randomly selected measurement points.

We have showcased the reconstruction results of multi-color objects employing the proposed methodology, with the validation scenario depicted in Fig. S18(a). Without requiring a hardware platform switch, CNN1 is trained to process low resolution fringe images as inputs and predict super-resolved background-free fringe amplitude images. The output numerator term (sine amplitude image) depicted in Fig. S18(b) verifies significant improvements in spatial resolution and fringe quality. Immediately following CNN1 conversion output, high-resolution wrapped phase is derived through the arctangent function as presented in Fig. S18(c). Figure S18(d) further demonstrates our ability to recover fine details such as curly hair and facial features on multi-colored head statues with precision while also elegantly reconstructing movement of palm texture details under different transient situations. It can be verified from Supplementary Video 8 that the proposed method still performs effective deciphering for multi-chromatic objects. Dynamic experimental results show that our proposed method can complete absolute 3D shape measurements of multiple objects with sharp edges, even when these objects are moving randomly.

We demonstrate the performance of the SSSR-FPP framework through high-resolution reconstruction of diverse complex samples comprising ceramics, foam, plastics, as shown in Fig. S18(e). In Fig. S18(f), we provide a visual representation of the reconstructed 3D point cloud data captured under different material conditions. To further validate the precision of our results, we conducted meticulous analysis by selecting ten specific points between plastic and ceramics objects. By perceiving feedback on the absolute difference between the measured values and the standard values, we can validate the depth reconstruction precision and distinguish the subtle differences among diverse materials. As depicted in Fig. S18(g), the disparity between measured points and their true values is confined within a mere 2%, underscoring the wide-ranging potential of SSSR-FPP in facilitating high-speed 3D imaging applications while paving new avenues in intelligent manufacturing and augmented reality.

## References


1. Luo, Y. *et al.* Computational imaging without a computer: seeing through random diffusers at the speed of light. *ELight* **2**, 4 (2022).
2. Hyun, C. M., Baek, S. H., Lee, M., Lee, S. M. & Seo, J. K. Deep learning-based solvability of underdetermined inverse problems in medical imaging. *Medical Image Analysis* **69**, 101967 (2021).
3. Nyquist, H. Thermal agitation of electric charge in conductors. *Physical Review* **32**, 110 (1928).
4. Ronneberger, O., Fischer, P. & Brox, T. U-net: Convolutional networks for biomedical image segmentation. In *International Conference on Medical Image Computing and Computer-Assisted Intervention*, 234–241 (Springer, 2015).
5. Chang, X., Bian, L. & Zhang, J. Large-scale phase retrieval. *Elight* **1**, 4 (2021).
6. Zuo, C., Huang, L., Zhang, M., Chen, Q. & Asundi, A. Temporal phase unwrapping algorithms for fringe projection profilometry: A comparative review. *Optics and Lasers in Engineering* **85**, 84–103 (2016).
7. Qian, J. *et al.* Deep-learning-enabled geometric constraints and phase unwrapping for single-shot absolute 3d shape measurement. *APL Photonics* **5** (2020).
8. He, K., Zhang, X., Ren, S. & Sun, J. Deep residual learning for image recognition. In *Proceedings of the IEEE Conference on Computer Vision and Pattern Recognition*, 770–778 (2016).
9. Zhang, Z. A flexible new technique for camera calibration. *IEEE Transactions on Pattern Analysis and Machine Intelligence* **22**, 1330–1334 (2000).
10. Feng, S. *et al.* General solution for high dynamic range three-dimensional shape measurement using the fringe projection technique. *Optics and Lasers in Engineering* **59**, 56–71 (2014).
11. Zheng, Y., Wang, S., Li, Q. & Li, B. Fringe projection profilometry by conducting deep learning from its digital twin. *Optics Express* **28**, 36568–36583 (2020).
12. Wang, F., Wang, C. & Guan, Q. Single-shot fringe projection profilometry based on deep learning and computer graphics. *Optics Express* **29**, 8024–8040 (2021).
13. Cai, J., Zeng, H., Yong, H., Cao, Z. & Zhang, L. Toward real-world single image super-resolution: A new benchmark and a new model. In *Proceedings of the IEEE/CVF International Conference on Computer Vision*, 3086–3095 (2019).
14. Hardie, R. C., Barnard, K. J., Bognar, J. G., Armstrong, E. E. & Watson, E. A. High-resolution image reconstruction from a sequence of rotated and translated frames and its application to an infrared imaging system. *Optical Engineering* **37**, 247–260 (1998).
15. Landau, H. Sampling, data transmission, and the Nyquist rate. *Proceedings of the IEEE* **55**, 1701–1706 (1967).
16. Yao, Y., Luo, Z., Li, S., Fang, T. & Quan, L. MVSNet: Depth inference for unstructured multi-view stereo. In *Proceedings of the European Conference on Computer Vision (ECCV)*, 767–783 (2018).

**17.** Mayya, V., Pai, R. M. & Pai, M. M. Automatic facial expression recognition using DCNN. *Procedia Computer Science* **93**, 453–461 (2016).

**18.** Feng, S. *et al.* Fringe pattern analysis using deep learning. *Advanced Photonics* **1**, 025001–025001 (2019).

**19.** Schonberger, J. L. & Frahm, J.-M. Structure-from-motion revisited. In *Proceedings of the IEEE Conference on Computer Vision and Pattern Recognition*, 4104–4113 (2016).

**20.** Durrant-Whyte, H. & Bailey, T. Simultaneous localization and mapping: part I. *IEEE Robotics & Automation Magazine* **13**, 99–110 (2006).

**21.** Seitz, S. M., Curless, B., Diebel, J., Scharstein, D. & Szeliski, R. A comparison and evaluation of multi-view stereo reconstruction algorithms. In *2006 IEEE Computer Society Conference on Computer Vision and Pattern Recognition (CVPR'06)*, vol. 1, 519–528 (IEEE, 2006).

**22.** Furukawa, Y., Hernández, C. *et al.* Multi-view stereo: A tutorial. *Foundations and Trends® in Computer Graphics and Vision* **9**, 1–148 (2015).
